# Impact of electrode orientation, myocardial wall thickness, and myofiber direction on intracardiac electrograms: numerical modeling and analytical solutions

**DOI:** 10.3389/fphys.2023.1213218

**Published:** 2023-07-10

**Authors:** Lore Leenknegt, Alexander V. Panfilov, Hans Dierckx

**Affiliations:** ^1^ Department of Mathematics, KU Leuven Campus KULAK, KU Leuven, Kortrijk, Belgium; ^2^ iSi Health–KU Leuven Institute of Physics-based Modeling for In Silico Health, KU Leuven, Leuven, Belgium; ^3^ Department of Physics and Astronomy, Ghent University, Ghent, Belgium

**Keywords:** bidomain, EGM, modeling, simulations, openCARP, solid angle

## Abstract

Intracardiac electrograms (iEGMs) are time traces of the electrical potential recorded close to the heart muscle. We calculate unipolar and bipolar iEGMs analytically for a myocardial slab with parallel myofibers and validate them against numerical bidomain simulations. The analytical solution obtained via the method of mirrors is an infinite series of arctangents. It goes beyond the solid angle theory and is in good agreement with the simulations, even though bath loading effects were not accounted for in the analytical calculation. At a large distance from the myocardium, iEGMs decay as 1/*R* (unipolar), 1/*R* ^2^ (bipolar and parallel), and 1/*R* ^3^ (bipolar and perpendicular to the endocardium). At the endocardial surface, there is a mathematical branch cut. Here, we show how a thicker myocardium generates iEGMs with larger amplitudes and how anisotropy affects the iEGM width and amplitude. If only the leading-order term of our expansion is retained, it can be determined how the conductivities of the bath, torso, myocardium, and myofiber direction together determine the iEGM amplitude. Our results will be useful in the quantitative interpretation of iEGMs, the selection of thresholds to characterize viable tissues, and for future inferences of tissue parameters.

## 1 Introduction

Heart rhythm disorders are still the main cause of death worldwide. The main tools to study cardiac arrhythmias and their properties non-invasively include measurements of electrical potentials in the body caused by electrical sources in the heart. There exist several mapping strategies, outlined by Choudhuri and Akhtar (2012). When recorded on the body surface, these signals are known as electrocardiograms (ECGs). To treat arrhythmias, a common method is intracardiac ablation (Bhaskaran et al., 2020), a procedure during which a catheter containing electrodes is inserted into the heart and the potentials are locally recorded, resulting in intracardiac electrograms (iEGMs). To guide the ablation, electro-anatomical voltage mapping (EAVM) is used, in which an electrode on a catheter roves over the cardiac wall and measures and maps the potential on a constructed mesh. Presently, only iEGMs have sufficient spatiotemporal resolutions to be used as the basis for reliable catheter ablation. To this purpose, cardiologists use spatial registration and processing packages, such as CardioInsight (Ramanathan and Jia, 2006) and EnSite (Ptaszek et al., 2018). Intracardiac electrograms (EGMs) are used to localize and characterize arrhythmia substrates from the amplitude, shape, and complexity of the signals (Bolick et al., 1986; Glashan et al., 2018).

The concrete motivation for this study results from ongoing debates in the medical literature concerning the interpretation of EGM properties. As a first example, Glashan et al. (2018) recently proposed that in order to delineate non-viable tissue regions based on the unipolar EGM amplitude [usually carried out using mapping strategies (Jackson et al., 2015) and classification rules (Marchlinski et al., 2000; Nayyar et al., 2018)], the total wall thickness should be taken into account. This is currently not the case, as medical standards prescribe constant thresholds of Φ_uni_ >1.5 mV for healthy tissues, and 0.5–1.5 mV for the border zone. Values Φ_uni_ <0.5 mV are considered to occur in the densely scarred myocardium (Marchlinski et al., 2000).

The aim of this study is to develop an analytical approach to explain and predict certain properties of the iEGM and to support these findings with simulation data.

**FIGURE 1:**
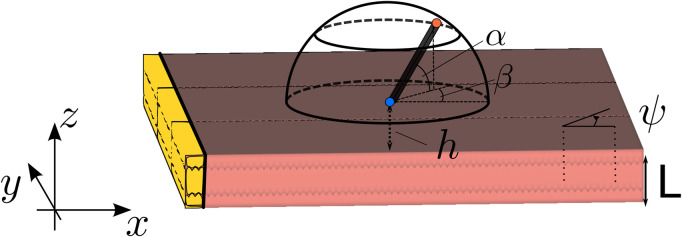
Setup of the simulation. The wave propagation is in all simulations in the X-direction. Here, *L* is the wall thickness, *ψ* is the angle between the myofiber direction and the direction of wave propagation, *α* is the elevation angle of the catheter, and *β* is the azimuthal angle. The interelectrode distance on the catheter is *d*, and *h* is the distance of the catheter tip to the myocardial wall. The highlighted (yellow) tissue is the stimulated tissue and, thus, the onset of the wave.

In brief, we considered an idealized geometry of the myocardial wall consisting of one layer with a constant myofiber direction, lying between spaces of constant conductivity, representing the cardiac cavity (blood pool) and the surrounding torso, see Figure 1. At distance *h* from the endocardium, we set two electrodes with interelectrode distance *d* to mimic a bipolar catheter, whose orientation is fixed by angles *α* and *β* in Figure 1. Our solution to bidomain equations (neglecting bath-loading effects) then yielded an explicit solution for the unipolar voltage Φ(*t*) registered by the catheter tip (distal electrode) and the bipolar signal, which equals the voltage difference across the electrode. The solution for the unipolar solution was found as an infinite series, see Supplementary Eq. 70. However, when the electrode was close to the endocardium, the first term in this summation already gave a reasonable approximation. Our main result can, thus, be stated as follows:

$$\Phi \left(t\right)\approx \frac{\left(\phi_{max}-\phi_{rest}\right)}{\pi }\frac{g_{\text{i},xx}}{\left(g_{\text{i},xx}+g_{\text{e},xx}+\eta g_{\text{B}}\right)}{\tilde{\Theta }}_{1}\left(t\right).$$ (1)

Here, *ϕ*_max_ − *ϕ*_rest_ is the difference in transmembrane voltage during upstroke (i.e., about 100 mV); *g*_i,*xx*_ and *g*_e,*xx*_ are the intra- and extracellular conductivities in the direction of wave propagation and *g*_i,*zz*_ and *g*_e,*zz*_ are the conductivities in the transmural direction. Together they determine the anisotropy ratio $\eta =\sqrt{\left(g_{\text{i},xx}+g_{\text{e},xx}\right)/\left(g_{\text{i},zz}+g_{\text{e},zz}\right)}$ that quantifies the anisotropy of the tissue. Finally, ${\tilde{\Theta }}_{1}$ is the angle subtended by the wave front when viewed from the electrode, after stretching the wall thickness over factor *η*.

The analytical result was confirmed by numerical bidomain simulations (Plank et al., 2021). From the novel description, we learned the explicit dependency of the uni- and bipolar iEGM on six conductivity parameters. Furthermore, the EGM amplitude was found to increase monotonically (with the saturation) with the wall thickness and to drastically reduce when the wave propagated perpendicular to the myofibers.

The remainder of this paper is structured as follows: Section 2 outlines the numerical methods. The analytical results are derived in Section 3, with the solution to the anisotropic case included in Supplementary Appendix A2. The analytical and numerical results were then used to assess the impact of the slab thickness, angle of incidence with the fibers, and electrode configuration (orientation, position, and interelectrode distance). Section 4 discusses our results and compares them to previous numerical and analytical works in the literature. Finally, a conclusion and outlook are given.

## 2 Methods

### 2.1 Physics model: bidomain equations

The wave propagation was modeled by bidomain equations (Tung, 1978; Roth, 1991; Clayton et al., 2011), which describe the evolution of the extracellular electrical potential $\Phi \left(\vec{r},t\right)$ , the intracellular electrical potential $\Phi_{\text{int}}\left(\vec{r},t\right)$ , and their difference, the transmembrane potential *V*_m_ = Φ_int_ − Φ. Within the myocardial wall, the following coupled partial differential equations are applied:

$$\vec{\nabla }\cdot \left(G_{\text{i}}+G_{\text{e}}\right)\vec{\nabla }\Phi =-\vec{\nabla }\cdot \left(G_{\text{i}}\vec{\nabla }V_{\text{m}}\right),$$ (2a)

$$\vec{\nabla }\cdot G_{\text{i}}\vec{\nabla }\left(V_{\text{m}}+\Phi \right)=\beta_{\text{M}}\left(C_{\text{M}}\frac{\partial V_{\text{m}}}{\partial t}+i_{\text{ion}}\left(V_{\text{m}},u\right)\right),$$ (2b)

$$\frac{\partial u}{\partial t}=K\left(V_{\text{m}},u\right).$$ (2c)

Here, **G**_i_ and **G**_e_ are the conductivity tensors for the intracellular and extracellular domain, *β*_M_ is the surface-to-volume ratio, *C*_M_ is the membrane capacitance, and *i*_ion_ describes the ion currents through the membrane. The ionic currents themselves depend on the transmembrane voltage and on ionic concentrations and membrane gates that are grouped in column vector **u**. The evolution of this state vector is governed by Eq. 2c. The functions *i*_ion_ and **K** together specify the reaction kinetics, i.e., the cardiac cell model (Clayton et al., 2011). In the bidomain model, the active myocardium is surrounded by a blood pool and torso, in which the electrical potential is denoted as Φ_*B*_ or Φ_*T*_. These regions only exhibit isotropic passive conduction.

$$\vec{\nabla }\cdot \left(g_{\text{B}}\vec{\nabla }\Phi_{B}\right)=0,\;\left(in\;blood\;pool\right)$$ (2d)

$$\vec{\nabla }\cdot \left(g_{\text{T}}\vec{\nabla }\Phi_{T}\right)=0.\;\left(in\;torso\right)$$ (2e)

Within our numerical study, we took *g*_B_ = *g*_T_, i.e., the myocardium was embedded in a bath of isotropic and homogeneous conductivity. In our analytical result, we used a three-layer geometry, with *g*_B_ ≠ *g*_T_. The myocardium there was a slab of thickness *L* parallel to the XY plane but was unbounded in the *X* and *Y* directions. At the outer boundary of the bath, Neumann boundary conditions were applied, which state that no electrical current can flow outside the bath. In our slab geometry, we applied the same boundary condition to the edge of the blood pool.

$$\vec{n}\cdot \vec{\nabla }\Phi_{T}=0,\;\left(at\;torso\;boundary\right)$$ (2f)

$$\vec{n}\cdot \vec{\nabla }\Phi_{B}=0.\;\left(at\;blood\;pool\;boundary\right)$$ (2g)

At the interface between the myocardium and blood pool and torso, the following transition conditions were applied: Φ is continuous across the interface, extracellular current flows to the bath, and intracellular current cannot flow to the bath. With * denoting the blood or torso domain, we have the following:

$$\Phi =\Phi_{*}\;\left(at\;myocardial\;boundary\right)$$ (2h)

$$\vec{n}\cdot \left(G_{\text{i}}+G_{\text{e}}\right)\vec{\nabla }\Phi =g_{*}\vec{n}\cdot \vec{\nabla }\Phi_{*},\;\left(at\;myocardial\;boundary\right)$$ (2i)

$$\vec{n}\cdot G_{\text{i}}\vec{\nabla }\Phi_{\text{int}}=0,\;(at\;myocardial\;boundary),$$ (2j)

where $\vec{n}$ is a normal vector to the interface (in either direction).

The unipolar electrical signal Φ was measured by a small electrode close to the heart at position ${\vec{r}}_{\text{electrode}}$ :

$$\Phi_{\text{unipolar}}\left(t\right)=\Phi \left({\vec{r}}_{\text{electrode}},t\right).$$ (3)

In experiments and clinical practice, physicians can also record the voltage difference between two electrodes. Inside the heart, convention is generally used to subtract the voltage of the proximal electrode (furthest away from the tissue) from the distal one (closest to the tissue). Thus, the bipolar EGM can be calculated as follows:

$$V_{\text{bipolar}}\left(t\right)=\Phi_{\text{prox}}\left(t\right)-\Phi_{\text{dist}}\left(t\right)=\Phi \left({\vec{r}}_{\text{prox}},t\right)-\Phi \left({\vec{r}}_{\text{dist}},t\right).$$ (4)

We now specify the electrode configuration used in our theoretical and numerical study.

### 2.2 Geometry set-up and electrode configuration

We locally considered the myocardial wall to be a slab of constant thickness *L*, such that the endocardial surface was located at the plane *z* = 0. The myocardial wall extended between *z* ∈ [−*L*, 0]. To obtain a propagating action potential in the XZ plane, a planar stimulus was applied at the left border of the medium (i.e., at most negative X-values in the domain). The relative position and orientation of the myocardial wall and sensing electrodes are depicted in Figure 1. The problem was assumed to be independent of the Y-coordinate (which is a simplification), but the Y-direction was nonetheless included in the numerical simulations.

At distance *h* above the endocardial surface (*z* = 0), a first (distal) electrode was placed, shown as the blue point in Figure 1, that can be used to acquire unipolar iEGMs, denoted as $\Phi_{\text{dist}}\left(t\right)=\Phi \left({\vec{r}}_{\text{dist}},t\right)$ .

At distance *d* from the first electrode, a second (proximal) electrode was placed (the red dot in Figure 1) to record $\Phi_{\text{prox}}\left(t\right)=\Phi \left({\vec{r}}_{\text{prox}},t\right)$ .

The catheter orientation was varied using two angles, shown in the study by Schuler et al. (2013): the azimuthal angle *β* ∈ [−90°, 90°] between the projection of the catheter on the tissue and the X-axis and the elevation angle *α* ∈ [0°, 180°] between the catheter and the tissue. In terms of these angles, the spatial vector pointing from the distal to the proximal electrode is given as follows:

$$\vec{d}=d\left(\cos\alpha \cos\beta {\vec{e}}_{x}+\cos\alpha \sin\beta {\vec{e}}_{y}+\sin\alpha {\vec{e}}_{z}\right).$$ (5)

To compare to the analytical results, we only used parallel myofibers within this study. We initiated plane waves along the X-direction of the slab and took myofibers within the XY plane, enclosing a constant angle *ψ* ∈ [0°, 90°] with the positive X-direction. Here, *g* ^*^_ij_ are the components of the tensor **G** ^*^ and * refers to either the intracellular or extracellular compartment; hence, we found the following:

$$\begin{array}{ccccc} g_{*,xx} & =g_{*,l}\cos^{2}\psi +g_{*,t}\sin^{2}\psi ,\\ g_{*,yy} & =g_{*,t}\cos^{2}\psi +g_{*,l}\sin^{2}\psi , & & & \\ g_{*,xy} & =2\left(g_{*,l}-g_{*,t}\right)\cos\psi \sin\psi , & \\ g_{*,zz} & =g_{*,t}, & \\ g_{*,xz} & =g_{*,yz}=0. \end{array}$$ (6)

Here, l and t stand for the longitudinal and transversal component, respectively. The angle *ψ* was used to quantify the effect of the angle between wave propagation and myofiber direction on iEGMs, which turned out to be an important characteristic of anisotropy.

### 2.3 Numerical solution to the bidomain equations

All the bidomain simulations were run using the open cardiac electrophysiology simulator for *in silico* experiments openCARP (Plank et al., 2021). The ten Tusscher–Panfilov 2006 model (TP06) with epicardial cells was used for reaction kinetics (ten Tusscher and Panfilov, 2006). The conductivities *g* used for the simulations (Plank et al., 2021) are *g*_i,l_ = 0.1527 S/m, *g*_i,t_ = 0.0312 S/m, *g*_e,l_ = 0.5485 S/m, *g*_e,t_ = 0.3361 S/m, and *g*_bath_ = 0.7 S/m. With these conductivity values, we measured a longitudinal CV of 0.42 m/s and a transverse CV of 0.2 m/s, which qualitatively agrees with the studies by Costa et al. (2013) and Caldwell et al. (2009). The bath-loading effects seen when using the values used by Bishop et al. (2011) and Bishop and Plank (2011b) are less prominent when using our values and are neglected in this study.

The simulations were performed on a 3D slab of size 70 × 70 ×*L* mm (see Figure 1), surrounded by a bath of 5 mm in all three directions. These tetrahedral meshes were generated using MeshTool (Neic et al., 2020) with a resolution of 0.2 mm.

The spatial discretization was carried out using Galerkin FEM (Sundnes et al., 2006). For temporal discretization, a parabolic solver was decoupled from an elliptic solver. The former uses the implicit Crank–Nicolson scheme (Fadugba et al., 2013), while the latter uses the direct forward Euler scheme. The time step size with which the partial differential equations were solved is equal to *dt* = 100 μs. A total time of 600 ms was simulated with a sampling step of 0.1 ms. A transmembrane current of strength 100 μA/cm^2^ with a duration of 1 ms was applied on the area of 1 × 70 × *L* mm (see Figure 1). A grounded region of 1 mm^3^ was added at the corner of the bath of (75, 75, *L* + 5) mm.

The simulations were run on an Intel^®^ Xeon^®^ Gold 6326 CPU @ 2.90 GHz. This machine has 64 threads, with two threads per core and 16 cores per socket, of which there are only two. Only 16 threads were used to run the simulations. Depending on the parameters, one simulation took between 9 h–13 h.

We report results for *h* = 1 mm, since for smaller values of *h*, this value becomes comparable to the finite element size and discretization artefacts affect the EGM amplitude. Furthermore, the lowest simulated interelectrode distance is *d* = 2 mm, which is the default value used in this paper.

Within each simulation, different electrode orientations were mimicked by recording the extracellular potential along 149 points regularly spaced on a hemisphere centered around the distal electrode position, similar to the study by Blauer et al., 2014; we took *α* ∈ {0°, 30°, 60°} and *β* ∈ { − 90°, −60°, …, 60°, 90°} and supplemented *α* = 90° (in which case *β* is irrelevant). This set was taken for three different interelectrode distances *d* ∈ {2, 3, 4} mm, resulting in 7 × 7 × 3 + 1 = 149 points, where “+1” is for the central distal electrode position at the center of the hemisphere (see blue dot in Figure 1).

We varied two quantities amongst the different *in silico* experiments: wall thickness *L* and wave-fiber angle *ψ*. The different values used for these parameters are *L*= 2, 5, and 10 mm and *ψ*= 0, 30, 60, and 90°. This set-up led to a total of 4*3 = 12 simulations, in which 149 unipolar EGMs were generated in each case.

### 2.4 Post-processing of the simulation results

The simulations provided us with the time course of the extracellular potential for the two points of the catheter. We studied the effect of the amplitude or peak-to-peak amplitude and the EGM width or duration, on the properties of the electrogram. The numerical simulations generated both depolarization (QRS complex) and repolarization (T-wave) parts. Both complexes could be selected by taking the first and second half of the signal, respectively. The analytical results only generated the depolarization wave. We compared iEGMs in terms of the following characteristics.

#### 2.4.1 Extremal values

Both minimum and maximum values of the electrical potential were calculated. The generated signals were smooth and densely sampled such that no pre-filtering was required.

#### 2.4.2 Peak-to-peak amplitude

Following clinical practice, we also report the peak-to-peak difference, Φ_pp_ = *ϕ*_max_ − *ϕ*_min_.

#### 2.4.3 Width of the EGM

The width of the EGM or EGM duration should reflect the time interval over which the peak is formed but is tedious to define in practice due to a vast variation in the signal morphology. Here, we defined the fraction of completion for both QRS and T waves, given as follows:

$$S\left(t\right)=\frac{\int_{-\infty }^{t}|\Phi \left(t\right)|dt}{\int_{-\infty }^{\infty }|\Phi \left(t\right)|dt}.$$ (7)

Then, in analogy to the measurement of the action potential duration, we define the start and stop time of the signal as follows: *S* (*t*_5_) = 0.05 and *S* (*t*_95_) = 0.95. Then, the time interval, including 90% of absolute surface variations under the signal curve, was as follows:

$$W_{90}=t_{95}-t_{5}.$$ (8)

This quantity was used in the following as the *width* of the EGM.

#### 2.4.4 The root-mean-squared error

To quantify the resemblance between two signals Φ_1_ and Φ_2_, we used the root-mean-squared error (RMSE) on the interval of interest *t* ∈ [*t*_0_, *t*_1_]:

$$\text{RMSE}=\sqrt{\frac{1}{t_{1}-t_{0}}\int_{t_{0}}^{t_{1}}|\Phi_{1}-\Phi_{2}{|}^{2}\text{d}t},$$ (9)

which yielded a value in mV.

## 3 Results

We first report an analytical solution for the iEGM in a slab with parallel fibers and then interpret the results.

### 3.1 Analytical solutions

The calculation of an EGM in an isotropic homogeneous medium is reviewed in Section 3.1.1 and is extended to a three-layered medium in Section 3.1.2 and improved even further to include the anisotropic case in Section 3.1.3.

#### 3.1.1 Analytical unipolar EGM in an isotropic homogeneous medium

The set of coupled Eq. 2a has not yet been solved analytically. However, if the distribution of the transmembrane potential $V_{m}\left(\vec{r},t\right)$ is known, Eq. 2a can be regarded as a Poisson problem:

$$-\vec{\nabla }\cdot \left(G_{\text{i}}+G_{\text{e}}\right)\vec{\nabla }\Phi =\rho \left(x,t\right),$$ (10)

with charge density

$$\rho =\vec{\nabla }\cdot \left(G_{\text{i}}\vec{\nabla }V_{\text{m}}\right).$$ (11)

A similar reasoning was used to approximate electrical signals from monodomain simulations in the pseudo-bidomain method or the lead-field method (Bishop and Plank, 2011a).

**FIGURE 2:**
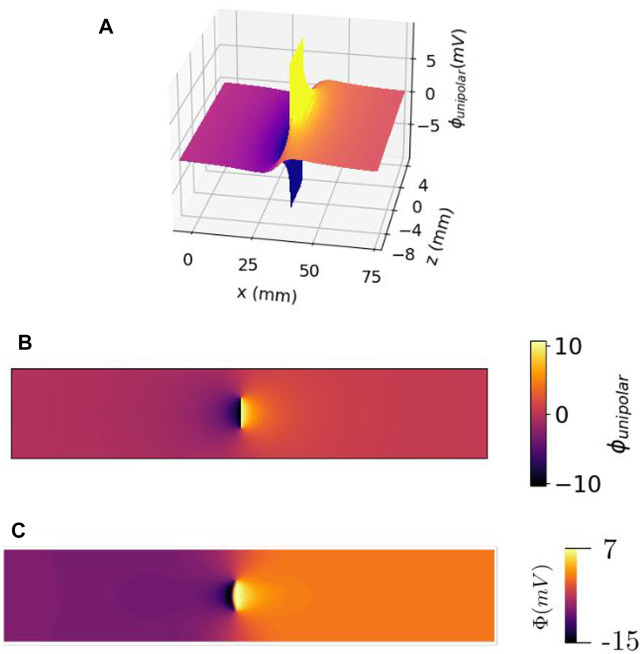
Electrical potential of a traveling wave within the cardiac wall, as a function of the lateral position (or time) *x* = *vt* and electrode distance from the myocardium *z*. **(A)** 3D view. **(B)** Top view of the same profile. It should be noted that there is no singularity of the potential, but a discontinuity (branch cut) at the wave front within the cardiac wall. **(C)** Same view as **(B)** but observed with simulated data.

The shape of the wave front in a slab with uniform fibers is in general non-planar, due to bath-loading effects; the presence of a conducting layer around the myocardium tends to accelerate the wave front near the tissue–bath interface (Roth, 1991; Bishop and Plank, 2011b; Bishop et al., 2011). In our simulation results with conductivity values taken from the literature, we found that the wave front was approximately planar, see Figure 2C. Therefore, within this study, we neglected bath loading effects and assumed that membrane potential *V*_m_ changed rapidly from resting state *ϕ*_rest_ = −86 mV to *ϕ*_max_ = +10 mV, over a plane parallel to the YZ plane. For a wave traveling to the positive X-direction, it was found that

$$V_{\text{m}}=\phi_{\text{max}}-\left(\phi_{\text{max}}-\phi_{\text{rest}}\right)H\left(x-vt\right),$$ (12)

with *H*(*x*) as the Heaviside step function.

By inserting this Ansatz (Eq. 12) into the bidomain of Eq. 2a, we obtained a Poisson problem (Eq. 10), with the following charge density:

$$\rho \left(x,t\right)=-\left(\phi_{max}-\phi_{rest}\right)g_{\text{i},xx}\delta^{\prime }\left(x-vt\right).$$ (13)

Here, *δ*(*x*) is the Dirac distribution that represents a localized charge (Arfken and Weber, 1995); its spatial derivative, as appearing in (Eq. 13), is the mathematical representation of an electrical dipole layer.

The general solution to this case could be obtained by linear superposition, in the form of Green’s functions. If $G\left(\vec{r},{\vec{r}}_{0}\right)$ was the electrical potential Φ created by a unit point charge at ${\vec{r}}_{0}$

$$-\vec{\nabla }\cdot \left(G_{\text{i}}+G_{\text{e}}\right)\vec{\nabla }\Phi =\delta \left(\vec{r}-{\vec{r}}_{0}\right),$$ (14)

respecting the boundary conditions, then the solution to (Eq. 10) is as follows:

$$\Phi \left(\vec{r},t\right)=∭G\left(\vec{r},{\vec{r}}_{0}\right)\rho \left({\vec{r}}_{0}\right)d^{3}r_{0}.$$ (15)

So, the search for an analytical solution came down to 1) finding the Green’s function and 2) integrating it over the source configuration. In our present scope, we only considered wave fronts parallel to the YZ plane, whence

$$\Phi \left(\vec{r},t\right)=\int_{-L}^{0}G\left(\vec{r},z_{0}\right)\rho \left(z_{0}\right)dz_{0}.$$ (16)

For general anisotropic and inhomogeneous media, the Green’s function has no closed form and should be computed numerically. In this paragraph, we continue the simplest case possible, where the conductivity in the Poisson’s Eq. 10 in the tissue is the same as in the blood and torso, in addition to being constant and isotropic. This implies that $G_{\text{i}}+G_{\text{e}}=g_{\text{B}}I=g_{\text{T}}I=\tilde{g}I$ , where we used $\tilde{g}$ to denote the simplified, lumped conductivity. We refer to this assumption as the “homogenized” case, since the differences between the conductivities of the myocardium, blood, and torso are neglected and have been replaced by a homogeneous conductivity $\tilde{g}$ . Our use of the term “homogenized” should not be confounded with the homogenization of the cellular structure within the tissue, which takes place during the derivation of bidomain equations (Neu and Krassowska, 1993).

In the analytical derivation, the bath was taken to be unbounded as opposed to the simulations. To denote that the potential too is an approximation, we indicated it as $\tilde{\Phi }$ .

Under this condition, the problem is reduced to a classical electrostatic problem:

$$\tilde{g}\Delta \tilde{\Phi }=\left(\phi_{max}-\phi_{rest}\right)g_{\text{i},xx}\delta^{\prime }\left(x-x_{0}\right)H\left(z+L\right)H\left(-z\right),$$ (17)

with the dipole sheet position, *x*_0_ = *vt*.

In the first step, we replaced the source by a line charge of unit strength parallel to Y that is placed at *x* = *x*_0_ and *z* = *z*_0_, i.e., *ρ* = *δ*(*x* − *x*_0_)*δ*(*z* − *z*_0_). Then, it was found from Gauss’ law that (Jackson, 1975)

$$\begin{array}{ll} \tilde{g}\Delta {\tilde{\Phi }}_{\text{line}} & =-\delta \left(x-x_{0}\right)\delta \left(z-z_{0}\right)\\ \Rightarrow {\tilde{\Phi }}_{\text{line}} & =-\frac{1}{2\pi \tilde{g}}\ln\sqrt{{\left(x-x_{0}\right)}^{2}+{\left(z-z_{0}\right)}^{2}}. \end{array}$$ (18)

Differentiating with respect to *x*_0_ gave the potential distribution of a dipole line, with the dipole oriented in the X-direction:

$$\begin{array}{ll} \tilde{g}\Delta {\tilde{\Phi }}_{\text{dipole line}} & =\left(\phi_{max}-\phi_{rest}\right)g_{\text{i},xx}\delta^{\prime }\left(x-x_{0}\right)\delta \left(z-z_{0}\right)\\ \Rightarrow {\tilde{\Phi }}_{\text{dipole line}} & =\frac{\left(\phi_{max}-\phi_{rest}\right)g_{i,xx}}{2\pi \tilde{g}}\frac{\left(x-x_{0}\right)}{{\left(x-x_{0}\right)}^{2}+{\left(z-z_{0}\right)}^{2}}. \end{array}$$ (19)

To obtain the potential generated by the wave front, the dipole line charges are needed to be stacked on top of each other in the Z-direction, *z*_0_ ∈ [−*L*, 0], where *z* = 0 corresponds to the endocardium.

$$\begin{array}{ll} {\tilde{\Phi }}_{\text{dipole layer}}\left(x,x_{0},z\right) & =\int_{-L}^{0}{\tilde{\Phi }}_{\text{dipole line}}\text{d}z_{0},\\ & =\frac{\left(\phi_{max}-\phi_{rest}\right)g_{i,xx}}{2\pi \tilde{g}}\int_{-L}^{0}\frac{x-x_{0}}{{\left(x-x_{0}\right)}^{2}+{\left(z-z_{0}\right)}^{2}}\text{d}z_{0},\\ & =\frac{\left(\phi_{max}-\phi_{rest}\right)g_{i,xx}}{2\pi \tilde{g}}\left(\text{arctan}\left(\frac{z+L}{x-x_{0}}\right)\right.\\ & \;-\left.\text{arctan}\left(\frac{z}{x-x_{0}}\right)\right), \end{array}$$ (20a)

$$\begin{array}{ll} & =\frac{\left(\phi_{max}-\phi_{rest}\right)g_{i,xx}}{2\pi \tilde{g}}\left(\text{arctan}\left(\frac{x-x_{0}}{z}\right)-\text{arctan}\left(\frac{x-x_{0}}{z+L}\right)\right). \end{array}$$ (20b)

**FIGURE 3:**
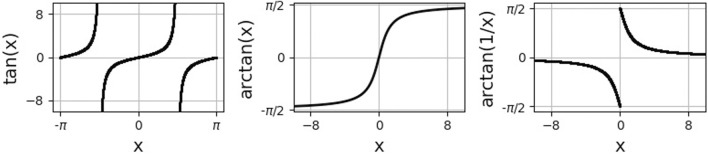
Trigonometric and inverse trigonometric functions used in the analytical calculations.

Here, arctan is the inverse of the tangent function on the interval (−*π*/2, *π*/2), see Figure 3. In the last step, we used that $\arctan\left(1/x\right)=\frac{\pi }{2}\mathrm{sgn}\left(x\right)-\arctan\left(x\right)$ . It should further be noted that arctan (−*x*) = − arctan(*x*).^1^

Since the wave profile propagated at constant speed *v* to the right, the wave front was located at *x*_0_(*t*) = *vt*, leading to the following spatiotemporal potential distribution:

$$\begin{array}{ll} \tilde{\Phi }\left(x,z,t\right) & ={\tilde{\Phi }}_{\text{dipole layer}}\left(x,vt,z\right)=\frac{\left(\phi_{max}-\phi_{rest}\right)g_{i,xx}}{2\pi \tilde{g}}\\ & \;\times \left(\arctan\left(\frac{x-vt}{z}\right)-\arctan\left(\frac{x-vt}{z+L}\right)\right). \end{array}$$ (21)

The potential registered by an electrode at position (*x*, *z*) = (0, *h*) is then the measured unipolar signal in the blood pool:

$$\begin{array}{ll} {\tilde{\Phi }}_{\text{unipolar}}\left(t\right) & ={\tilde{\Phi }}_{\text{dipole layer}}\left(0,vt,h\right)=\frac{\left(\phi_{max}-\phi_{rest}\right)g_{i,xx}}{2\pi \tilde{g}}\\ & \;\times \left(-\arctan\left(\frac{vt}{h}\right)+\arctan\left(\frac{vt}{h+L}\right)\right). \end{array}$$ (22)

**FIGURE 4:**
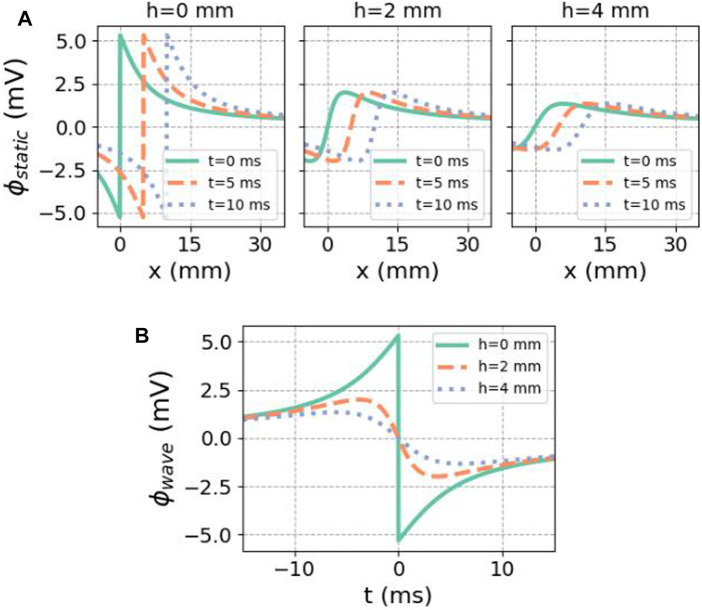
Analytical solution for the potential caused by a finite traveling dipole sheet in the X-direction. **(A)** Spatial potential profile at different distances *h* to the myocardium, for subsequent time steps. In the direction of front propagation (positive X), the potential is elevated. **(B)** Regarded as a function of time at a fixed recording position, there is first an upward deflection in the unipolar signal. In both cases, we used *ϕ*_max_ = 30 mV, *ϕ*_min_ = −86 mV, *g*_i,*xx*_ = 0.1527 S/m, $\tilde{g}=0.8341$ S/m, *L* = 5 mm, and *v* = 1 mm/ms.

Snapshots of spatial profiles at different times and different distances *h* to the endocardial surface are shown in Figure 4. When the signal is recorded on the endocardial surface, one can set $h\overset{>}{\to }0$ in (Eq. 22), such that for an electrode at x = 0:

$${\tilde{\Phi }}_{\text{unipolar}}\left(t\right)=-\frac{\left(\phi_{max}-\phi_{rest}\right)g_{i,xx}}{2\pi \tilde{g}}\arctan\left(\frac{L}{vt}\right),$$ (23)

which first entails an upward deflection, followed by a finite downward jump at *t* = 0 and a restoring phase, see Figure 4A.

**FIGURE 5:**
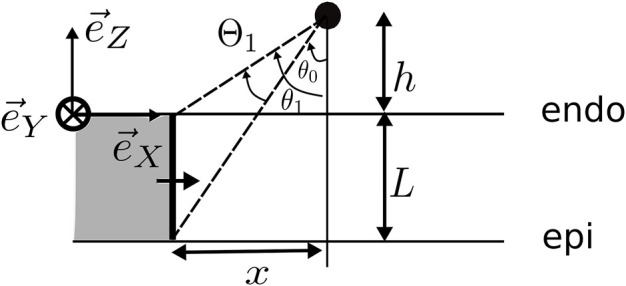
Interpretation of the arctan(*x*) and solid angles in the myocardium.

Figure 5 shows how this result can be interpreted geometrically. Since $\tan\left(\theta_{0}\right)=\frac{vt}{h}$ and $\tan\left(\theta_{1}\right)=\frac{vt}{h+L}$ , the result can be written in terms of the angle Θ_1_ = *θ*_1_ − *θ*_0_ under which the wave front is seen as follows:

$${\tilde{\Phi }}_{\text{unipolar}}\left(t\right)=\frac{\left(\phi_{max}-\phi_{rest}\right)g_{i,xx}}{2\pi \tilde{g}}\Theta_{1}.$$ (24)

This result is in agreement with the classical solid angle theory for the electrogram (Bayley and Berry, 1964; Holland and Arnsdorf, 1977; Van Oosterom, 2002; Macfarlane et al., 2010). The angle *θ* from Figure 5 can be extended with the angle *ξ* that a point on the wave front makes with respect to the Y-axis. Then, *ξ* and *θ* are spherical coordinates centered on the Y-axis, which obey dΩ = d*θ*d*ξ* sin *ξ*. Since we worked with a slab geometry, the angle *ξ* under which the wave front is seen always extends from 0 to *π*, whence

$$\Omega \left(\theta_{0},\theta_{1}\right)=\iint d\Omega =\int_{0}^{\pi }d\xi \int_{\theta_{0}}^{\theta_{1}}d\theta \sin\xi \;=2\left(\theta_{1}-\theta_{0}\right)=2\Theta_{1}.$$ (25)

Hence, doubling the planar angles Θ in this work will give the corresponding solid angle in 3D. It should be noted that angles Θ are expressed in radians, while the other angles used in this work are reported in degrees.

Thus, in the homogenized approximation, the unipolar EGM is proportional to the angle Θ_1_ subtended by the wave front when viewed from the electrode at any given time. The potential difference *ϕ*_max_ − *ϕ*_rest_ can be measured in experiments and has a value of around 120 mV (Plonsey, 1965; Plonsey, 1974; Holland and Arnsdorf, 1977). The value of *g*_i,*xx*_ can also be measured.

However, in the homogenized theory, there is no fixed rule to estimate the lumped conductivity $\tilde{g}$ as a weighted average of the conductivities in the problem: *g*_i,l_, *g*_i,t_, *g*_e,l_, *g*_e,t_, *g*_B_, and *g*_T_. In the following, we showed the relation between them from the exact analytical solution in the case with and without anisotropy.

#### 3.1.2 Analytical unipolar EGM in an isotropic three-layered geometry

Analytical calculations of potentials generated by dipole charges in inhomogeneous media representing the heart have been carried out before (Bayley and Berry, 1964; Kempner and Grayzel, 1970; Plonsey, 1974). However, these are focused on ECG generation and, therefore, adopted a circular geometry representing a cross-section through the heart.

In this paragraph, we incorporated the different conductivities of the blood (*g*_B_), myocardium (*g*_M_, given by *g*_i,*xx*_ + *g*_e,*xx*_), and torso (*g*_T_), for now assuming that the myocardium is an isotropic layer of thickness *L*. The mathematical problem is similar to finding the electrostatic potential in a three-layered medium with different electrical permittivities. For two layers, the result was detailed in the study by Jackson (1975) using the method of mirror charges, and it was used by Plonsey and Barr (1987) to obtain potentials inside the myocardium. The case where the two outer layers have equal properties and the recording is made in the middle layer has also been described in a study of quantum dots (Escribano et al., 2017).

In case of a three-layered medium, the solution method came down to reflecting the position of the sources within the myocardium on the other side of myocardium–torso and myocardium–blood interfaces and solving the interface conditions to recursively find all the strengths of the mirror sources.

This procedure is outlined in our Supplementary Appendix A1. We chose Cartesian coordinates, such as *z* > 0, which represents the blood pool, −*L* < *z* < 0 the (isotropic) myocardium and *z*< −*L* the torso domain (assumed homogeneous).

The factors relating to the strengths of different mirror sources are, with * equal to B (blood) or T (torso):

$$h_{*}=\frac{g_{\text{M}}-g_{*}}{g_{\text{M}}+g_{*}},\;v_{*}=\frac{2g_{*}}{g_{\text{M}}+g_{*}}.$$ (26)

Solving the recursion relation then led to the following explicit series solution:

$$\begin{array}{ll} \Phi_{\text{B}}\left(x,z,t\right) & =\frac{v_{\text{B}}g_{\text{M}}\left(\phi_{max}-\phi_{rest}\right)}{2\pi g_{\text{B}}}\overset{\infty }{\underset{j=0}{\sum }}h_{\text{B}}^{\left\lfloor \frac{j}{2}\right\rfloor }h_{\text{T}}^{\left\lceil \frac{j}{2}\right\rceil }\times \left[\arctan\left(\frac{x-vt}{z+jL}\right)\right.\\ & \;-\left.\arctan\left(\frac{x-vt}{z+\left(j+1\right)L}\right)\right], \end{array}$$ (27a)

$$\;=\frac{v_{\text{B}}g_{\text{M}}\left(\phi_{max}-\phi_{rest}\right)}{2\pi g_{\text{B}}}\overset{\infty }{\underset{j=0}{\sum }}h_{\text{B}}^{\left\lfloor \frac{j}{2}\right\rfloor }h_{\text{T}}^{\left\lceil \frac{j}{2}\right\rceil }\Theta_{j+1}.$$ (27b)

We observed that including inhomogeneous conductivities led to focusing and defocusing of electrical field lines [see (Jackson, 1975)], which can alternatively be interpreted as reflections of the source at the endo- and epicardial boundaries.

Although the result was formulated using the angle Θ, Eq. 27 go beyond the solid angle theory, as the solid angles subtended by the reflection are also included. Considering only the first term of the expansion led to $\Phi_{\text{B}}\left(x,z,t\right)\approx -\frac{v_{\text{B}}g_{\text{M}}\left(\phi_{max}-\phi_{rest}\right)}{2\pi g_{\text{B}}}\Theta_{1}$ . A comparison with the homogenized result (Eq. 24) suggested that $\tilde{g}=\frac{g_{\text{B}}}{v_{\text{B}}}=\frac{g_{\text{M}}+g_{\text{B}}}{2}$ . Thus, in the homogenized approach, the chosen effective conductivity should be equal to the arithmetic mean of myocardial and blood conductivities. This result was refined in the following paragraph to also include anisotropy.

#### 3.1.3 Analytical unipolar EGM in an anisotropic three-layered geometry

To add anisotropy of the myocardium to the derivation, we denoted *g*_M_ = *g*_i,*xx*_ + *g*_e,*xx*_ and *g*_*zz*_ = *g*_i,*zz*_ + *g*_e,*zz*_. To reuse the isotropic solution from the previous paragraph, we restored isotropy in the myocardial domain by re-scaling the *Z*-axis according to the square root of the conductivity ratio (see Figure 8), inspired by previous work on cardiac anisotropy (Wellner et al., 2002; Verschelde et al., 2007; Young and Panfilov, 2010). Thus, we used a (dimensionless) re-scaling factor, which also appeared in the study by Plonsey and Barr (1987):

$$\eta =\sqrt{\frac{g_{\text{M}}}{g_{zz}}}=\sqrt{\frac{g_{\text{i},xx}+g_{\text{e},xx}}{g_{\text{i},zz}+g_{\text{e},zz}}}.$$ (28)

For *η* = 1, the configuration is that of three parallel layers described previously.

For *η* ≠ 1, we defined a re-scaled *Z*-coordinate as follows:

$$Z=\left\{\begin{array}{cc} z+L-\eta L,\; & if\;z<-L\\ \eta z,\; & if\;-L\leq z\leq 0\\ z.\; & if\;0<z \end{array}\right.$$ (29)

In the following, we took the re-scaled tissue thickness to be *ℓ* = *ηL*. In Supplementary Appendix A2, it was verified that this reduced the problem to the one with three homogeneous layers, but at the interface conditions, *g*_M_ needed to be replaced by *g*_M_/*η*. Anisotropy, thus, affects three elements. First, the transfer coefficients are changed to the following:

$$H_{*}=\frac{g_{\text{M}}-\eta g_{*}}{g_{\text{M}}+\eta g_{*}},\;V_{*}=\frac{2g_{*}}{g_{\text{M}}+\eta g_{*}}.$$ (30)

Second, the effective tissue thickness becomes *ℓ* = *ηL*, meaning that the myocardium appears to be thicker, for wave propagation along the myofiber direction (as then, both *g*_*xx*_ and *η* increase). The geometric re-scaling also affects the solid angles, which we will denote as $\tilde{\Theta }$ here. For unipolar signal scales in the linear leading order with Θ_1_, we expect that maximal signal amplitude is reached for the case *ψ* = 0, i.e., wave propagation parallel to the myofibers. Third, wave speed *v* also depends on the myofiber orientation.

At the end of our derivation, the electrical potential measured in the blood is as follows:

$$\begin{array}{ll} \Phi_{\text{B}}\left(x,z,t\right) & =\frac{V_{\text{B}}g_{\text{i},xx}\left(\phi_{max}-\phi_{rest}\right)}{2\pi g_{\text{B}}}\overset{\infty }{\underset{j=0}{\sum }}H_{\text{B}}^{\left\lfloor \frac{j}{2}\right\rfloor }H_{\text{T}}^{\left\lceil \frac{j}{2}\right\rceil }\times \left[\arctan\left(\frac{x-vt}{z+j\eta L}\right)\right.\\ & \;-\left.\arctan\left(\frac{x-vt}{z+\left(j+1\right)\eta L}\right)\right], \end{array}$$ (31a)

$$=\frac{V_{\text{B}}g_{\text{i},xx}\left(\phi_{max}-\phi_{rest}\right)}{2\pi g_{\text{B}}}\overset{\infty }{\underset{j=0}{\sum }}H_{\text{B}}^{\left\lfloor \frac{j}{2}\right\rfloor }H_{\text{T}}^{\left\lceil \frac{j}{2}\right\rceil }{\tilde{\Theta }}_{j+1}.$$ (31b)

Equation 31 is the main analytical result of this paper. It goes beyond the solid angle theory, as it uses re-scaled angles due to anisotropy and takes into account several mirror layer reflections, due to the inhomogeneity of the layers, see Figure 8. Keeping only the first term in (Eq. 31), we get the following:

$$\begin{array}{ll} \phi_{\text{B}}^{\text{one}}\left(X,Z\right) & \approx \frac{g_{\text{i},xx}\left(\phi_{max}-\phi_{rest}\right)V_{\text{B}}}{2\pi g_{\text{B}}}{\tilde{\Theta }}_{1},\\ & =\frac{\left(\phi_{max}-\phi_{rest}\right)}{\pi }\frac{g_{\text{i},xx}}{\left(g_{\text{M}}+\eta g_{\text{B}},,\right)}{\tilde{\Theta }}_{1}=A{\tilde{\Theta }}_{1}. \end{array}$$ (32)

Here, we introduced the notation *A* as the proportionality factor between the electrical potential measurement and the angle. This prefactor depends on the conductivities and fiber orientation angle *ψ*. If one would furthermore neglect the re-scaling in the angles (i.e., $\Theta_{j}\approx {\tilde{\Theta }}_{j}$ , if *η* ≈ 1), one recovers (Eq. 24) with the homogenized conductivity being equal to an anisotropy-weighted average:

$$\tilde{g}=\frac{g_{\text{B}}}{V_{\text{B}}}=\frac{g_{\text{M}}+\eta g_{\text{B}}}{2},$$ (33)

with anisotropy ratio *η* in the direction of wave propagation given by (Eq. 28).

#### 3.1.4 Analytical solution for bipolar iEGMs

Bipolar signals are the difference of unipolar signals, see (Eq. 4).

For the homogenized case, from (Eq. 23), we found, with p used for the proximal and d for the distal electrode positions:

$$\begin{array}{ll} V_{\text{bipolar}}\left(t\right) & =\Phi_{\text{unipolar}}\left(x_{\text{d}},z_{\text{d}},t\right)-\Phi_{\text{unipolar}}\left(x_{\text{p}},z_{\text{p},t}\right)\\ & =\frac{\left(\phi_{max}-\phi_{rest}\right)g_{i,xx}}{2\pi \tilde{g}}\left[\arctan\left(\frac{x_{\text{d}}}{z_{\text{d}}}\right)-\arctan\left(\frac{x_{\text{d}}}{z_{\text{d}}+L}\right)\right.\\ & \;\left.+\;\arctan\left(\frac{x_{\text{p}}}{z_{\text{p}}+L}\right)-\arctan\left(\frac{x_{\text{p}}}{z_{\text{p}}}\right)\right]. \end{array}$$ (34)

Inserting the electrode coordinates *x*_d_ = *x*_0_ − *vt*, *z*_d_ = *h*, and *x*_p_ = *x*_0_ + *d* cos *β* cos *α* − *vt*, *z*_p_ = *h* + *d* sin *α* yielded the following:

$$\begin{array}{ll} V_{\text{bipolar}}\left(t\right) & =\frac{\left(\phi_{max}-\phi_{rest}\right)g_{i,xx}}{2\pi \tilde{g}}\left[\arctan\left(\frac{x_{0}-vt}{h}\right)-\arctan\left(\frac{x_{0}-vt}{h+L}\right)\right.\\ & \;+\arctan\left(\frac{x_{0}+d\cos\beta \cos\alpha -vt}{h+d\sin\alpha +L}\right)\\ & \;-\left.\arctan\left(\frac{x_{0}+d\cos\beta \cos\alpha -vt}{h+d\sin\alpha }\right)\right]. \end{array}$$ (35)

If the catheter was held parallel to the wave propagation direction or perpendicular to the endocardium, we get the following expressions:

$$\begin{array}{ll} V_{\text{bip},\|}\left(t\right) & =\frac{\left(\phi_{max}-\phi_{rest}\right)g_{i,xx}}{2\pi \tilde{g}}\left[\arctan\left(\frac{x_{0}-vt}{h}\right)-\arctan\left(\frac{x_{0}-vt}{h+L}\right)\right.\\ & \;\left.+\;\arctan\left(\frac{x_{0}+d-vt}{h+L}\right)-\arctan\left(\frac{x_{0}+d-vt}{h}\right)\right],\\ V_{\text{bip},⊥}\left(t\right) & =\frac{\left(\phi_{max}-\phi_{rest}\right)g_{i,xx}}{2\pi \tilde{g}}\left[\arctan\left(\frac{x_{0}-vt}{h}\right)-\arctan\left(\frac{x_{0}-vt}{h+L}\right)\right.\\ & \;\left.+\;\arctan\left(\frac{x_{0}-vt}{h+d+L}\right)-\arctan\left(\frac{x_{0}-vt}{h+d}\right)\right]. \end{array}$$ (36)

**FIGURE 6:**
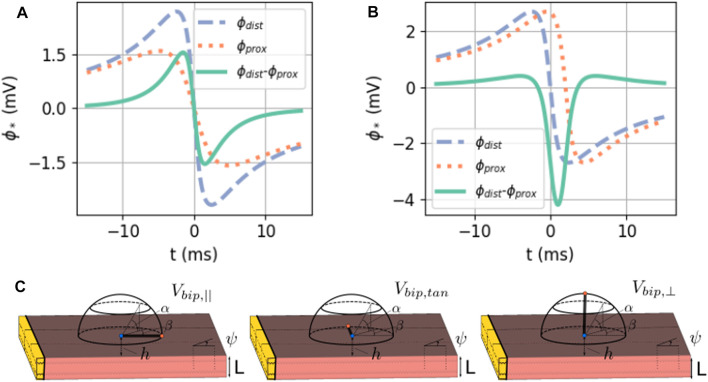
**(A)** Exact solution of the bipolar signal as a local potential difference. Here, *ϕ*_max_ = 30 mV, *ϕ*_min_ = −86 mV, *g*_i,*xx*_ = 0.1527 S/m, $\tilde{g}=0.8341$ S/m, *L* = 5 mm, and *v* = 1 mm/ms. The green solid line denotes *V*_bip,⊥_ in **(A)** and *V*_bip,‖_ in **(B)**. **(C)** Three main measuring directions of the bipolar extracellular potential *V*_bipolar_.

The bipolar signals in the main directions are shown as a difference of unipolar signals in Figure 6.

From the analytical result in case of anisotropy (Eq. 31), an exact solution for the bipolar electrogram can be found, see Supplementary Appendix A3.

#### 3.1.5 Analytical model for the wave speed

The only remaining unknown parameter in our analytical solution is the wave speed *v*. This speed depends on the local cell kinetics, i.e., the term *i*_ion_ in bidomain equations. Within this study, we neglected the bath-loading effect, such that the wave became planar. Its propagation velocity will then depend on the conductivities in the X-direction and was, therefore, affected by *g*_i, *xx*_, *g*_e, *xx*_, *g*_B_, and *g*_T_. In one spatial dimension, the intra- and extracellular conductivity tensors are numbers and are, hence, proportional to each other, under which condition the bidomain model could be simplified to a monodomain description (Bishop et al., 2011; Clayton et al., 2011). The resulting diffusion constant equals

$$D=\frac{1}{\beta_{m}C_{m}}\frac{g_{\text{i},xx}g_{\text{e},xx}}{g_{\text{i},xx+g_{\text{e},xx}}}.$$ (37)

Through the dimensional analysis of the monodomain equation, it was found that the conduction velocity is proportional to $\sqrt{D}$ , whence

$$v\left(\psi \right)=v_{\|}{\left(\frac{g_{\text{i,l}}+g_{\text{e,l}}}{g_{\text{i,l}}g_{\text{e,l}}}\right)}^{1/2}{\left[\frac{\left(g_{\text{i,l}}\cos^{2}\psi +g_{\text{i,t}}\sin^{2}\psi \right)\left(g_{\text{e,l}}\cos^{2}\psi +g_{\text{e,t}}\sin^{2}\psi \right)}{\left(\left(g_{\text{e,l}}+g_{\text{i,l}}\right)\cos^{2}\psi +\left(g_{\text{e,t}}+g_{\text{i,t}}\right)\sin^{2}\psi \right)}\right]}^{1/2}.$$ (38)

This reasoning is held for the bulk of a bidomain region, neglecting the bath-loading effects.

From simulations with *ψ* = 0° or 90°, we measured that *v*_‖_ = 0.42 m/s. This value was inserted into the following analytical solutions to have no more free parameters in the theory.

### 3.2 Comparison between analytical and numerical solutions for iEGMs

#### 3.2.1 Difference between analytical approximations

**FIGURE 7:**
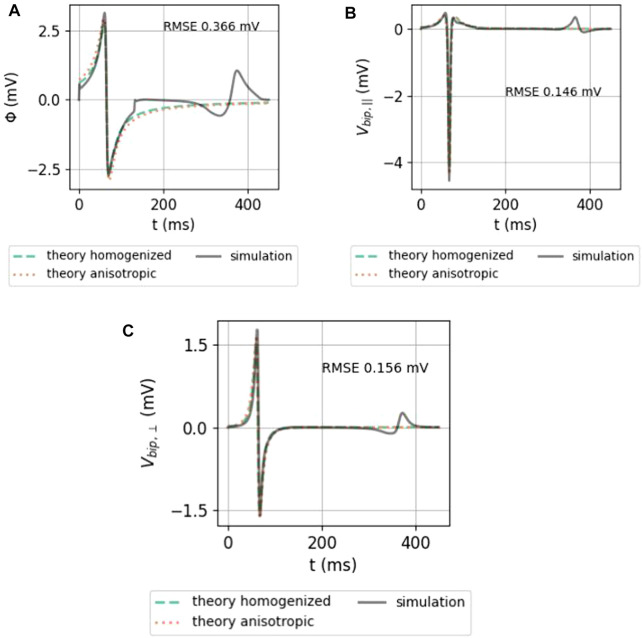
Comparison between the analytical numerical solution to the EGM near a slab with wave propagation along the myofiber direction. **(A)** Unipolar signal (gray), showing a far-field artefact in the simulation result, occurring since the wave front hits the end of the myocardial slab. **(B)** Bipolar signal measured parallel to the myocardial surface in the direction of wave propagation. **(C)** Bipolar signal measured perpendicular to the myocardial wall. The slab thickness was *L* = 5 mm. Furthermore, *ϕ*_max_ = 30 mV, *ϕ*_min_ = −86 mV, *g*_i,*xx*_ = 0.1527 S/m, $\tilde{g}=0.8341$ S/m, *v* = 0.42 mm/ms, and *d* = 2 mm. Wave speed *v* is the only free parameter in the theory and was measured in the simulation. The RMSE (Eq. 9) between the simulated signal and the anisotropic theoretical prediction was computed between 0 and 250 ms.

In Figure 7, the analytical approximations (homogenized, green solid line and anisotropic, dotted orange line) are compared to the simulated unipolar and bipolar signal in the two main directions. The repolarization (T-wave) was not included in the theoretical framework here and is, therefore, absent in theory graphs.

Both homogenized and anisotropic solutions follow the same qualitative behavior, and the amplitudes are in the correct range. When looked at more closely, it can be seen that the amplitudes of the anisotropic solution agree slightly better than the homogenized approximation. The difference, however, is small. The RMSE (Eq. 9) of the anisotropic solution with the simulated signal is shown on each plot.

**FIGURE 8:**
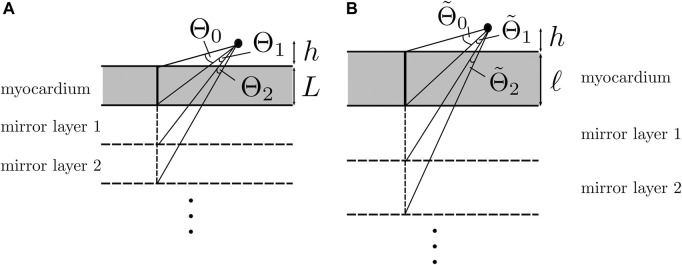
Solid angle contributions caused by mirror sources. **(A)** Isotropic three-layered medium. **(B)** Anisotropic three-layered medium, after re-scaling of the *Z*-coordinate. If $\frac{h}{L}\ll 1$ , then the first term of the series offers a good approximation.

**FIGURE 9:**
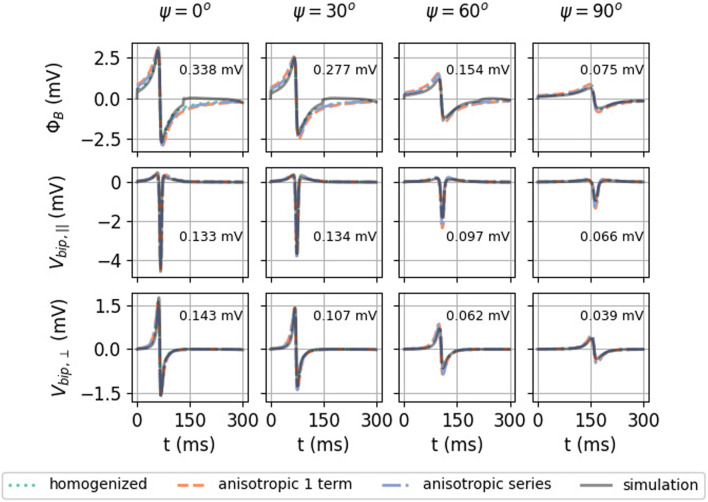
Effect of myofiber direction on the EGM amplitude: homogenized (see Eqs 23, 33), re-scaled anisotropic (one term) (see Eq. 32), and full series solution (see Eq. 31). Solutions shown here have *h* = 1 mm, *L* = 5 mm, and *d* = 2 mm. In every plot, the RMSE (Eq. 9) was calculated between 0 and 300 ms between the simulated signal and the anisotropic series prediction.

To understand why keeping the first terms in the expansion works, let us consider the relative importance of the first mirrored layer (Θ_2_) to the myocardium (Θ_1_). We found that Θ_2_ ≪Θ_1_ during wave passing if the electrode was close to the tissue (*h* ≪ *ηL*), see Figure 8. Thus, we expect the best convergence and a good approximation when the electrode is closer to the tissue, where the tissue is thick and *η* is large, i.e., for wave propagation along the myofiber direction. The latter can be seen in the top row of Figure 9, where the unipolar voltage (Φ_B_ or Φ_uni_) is shown. The higher the *ψ* value, the more the analytical solution differs from the simulated curve.

#### 3.2.2 iEGMs for different myofiber orientations

In Figure 9, the homogenized isotropic theory is plotted together with the inhomogeneous anisotropic theory (using only one or three terms). Strikingly, the amplitude of the EGM decreases significantly if the wave propagates perpendicular to the myofibers. For example, for unipolar potential Φ_*B*_, the peak-to-peak amplitude changes from 4.64 mV at *ψ* = 0° to 1.27 mV at *ψ* = 90°, implying a reduction factor of 3.6.

#### 3.2.3 iEGMs for different catheter orientations

**FIGURE 10:**
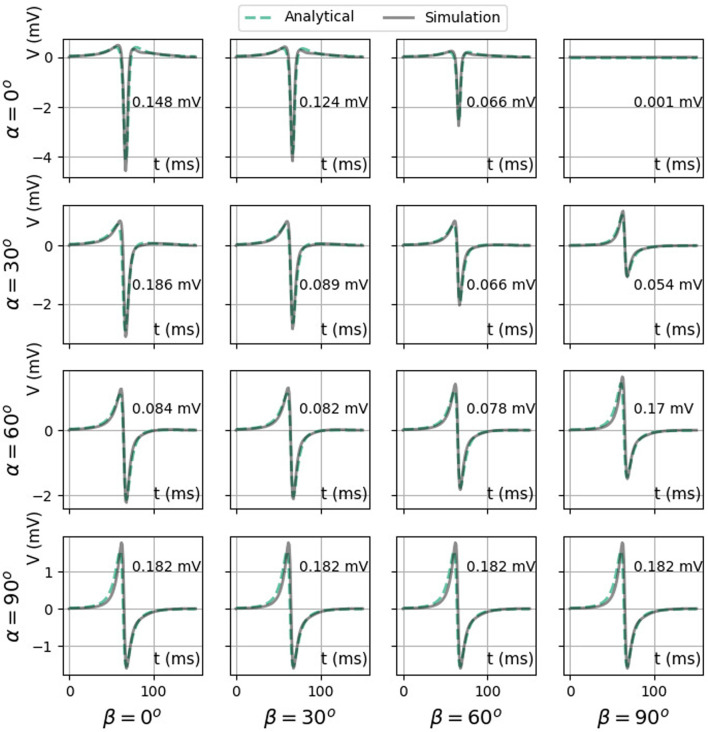
Comparison between two analytical bipolar signals as a function of time: exact difference between two unipolar signals (Eq. 35) (solid color) and the simulated signal (solid gray line). Here, we used *ϕ*_max_ = 30 mV, *ϕ*_min_ = −86 mV, *g*_i,*xx*_ = 0.1527 S/m, $\tilde{g}=0.8341$ S/m, *L* = 5 mm, *v* = 0.42 mm/ms, and *d* = 2 mm. The RMSE (Eq. 9) between the two signals is shown on every plot, calculated between 0 and 150 ms.

Figure 10 shows simulated and theoretical bipolar signals for different catheter orientation angles *α* and *β*, as defined in Figure 1. The case *α* = *β* = 0° (top left panel) corresponds to a bipolar electrode directed parallel to the wave propagation and shows a signal equal to *V*_bip,‖_(*t*), see (Eq. 36). This signal is even and has two zeros (see Figure 6B) and three extrema. Similarly, a bipolar electrode directed normally toward the endocardium (*α* = 90° for any *β* value) yields *V*_bip,⊥_(*t*) (Eq. 36), a signal with uneven symmetry (see Figure 6C and bottom row of Figure 10). This signal has only one zero crossing and vanishes for *x* − *vt* = ±*∞*. For intermediate orientations, the result gradually evolves from *V*_bip,‖_ to *V*_bip,⊥_. In all cases, it is well represented by the analytical solution.

#### 3.2.4 Bipolar iEGMs for different distances to the endocardium

**FIGURE 11:**
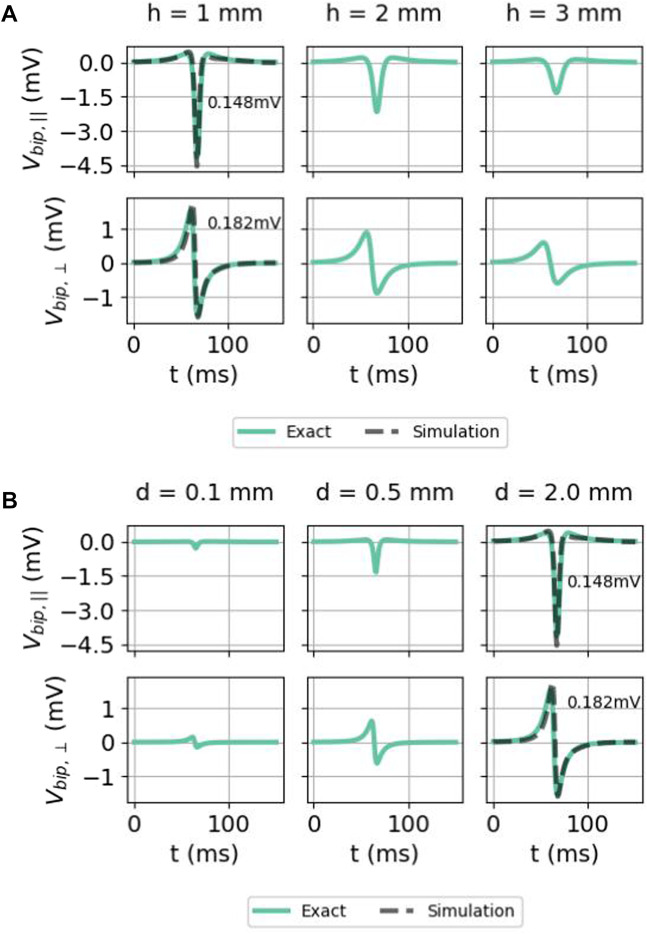
Effect of **(A)** the distance of the electrode to endocardium *h* and **(B)** interelectrode distance *d* on bipolar electrogram signals in the isotropic homogeneous medium. Here again, the two signals were given as follows: exact difference between two unipolar signals (Eq. 35) (solid colored) and the simulated signal (solid gray) (only for *h* = 1 mm and *d* = 2 mm). The comparison was carried out both for the parallel and normal electrical field component. Here, we used *x* = 0 mm, *ϕ*_max_ = 30 mV, *ϕ*_min_ = −86 mV, *g*_i,*xx*_ = 0.1527 S/m, $\tilde{g}=0.8341$ S/m, *L* = 5 mm, and *v* = 0.42 mm/ms. For parameters for which there is simulated data, the RMSE (Eq. 9) is given on the plot, calculated between 0 and 150 ms.

The effect of measuring further away from the cardiac wall (higher *h*) is shown in Figure 11A. At a larger distance, the maximal solid angle subtended Θ_0_ or ${\tilde{\Theta }}_{0}$ becomes smaller, such that the amplitude of the signal is reduced.

#### 3.2.5 Bipolar iEGMs for different interelectrode distances

Bipolar signals for different interelectrode distances *d* are shown in Figure 11B. For both orientations, the signal amplitude grows with the increased interelectrode distance. This can be understood by a first-order Taylor approximation around the distal electrode:

$$V_{\text{bip}}\left(t\right)=\Phi \left({\vec{r}}_{\text{dist}}\right)-\Phi \left({\vec{r}}_{\text{dist}}+\vec{d}\right)\approx -\vec{\nabla }\Phi \left({\vec{r}}_{\text{dist}}\right)\cdot \vec{d},$$ (39)

which grows linearly with the interelectrode distance $d=\|\vec{d}\|$ .

### 3.3 Interpretation of EGM characteristics

#### 3.3.1 Extraction of EGM characteristics

**FIGURE 12:**
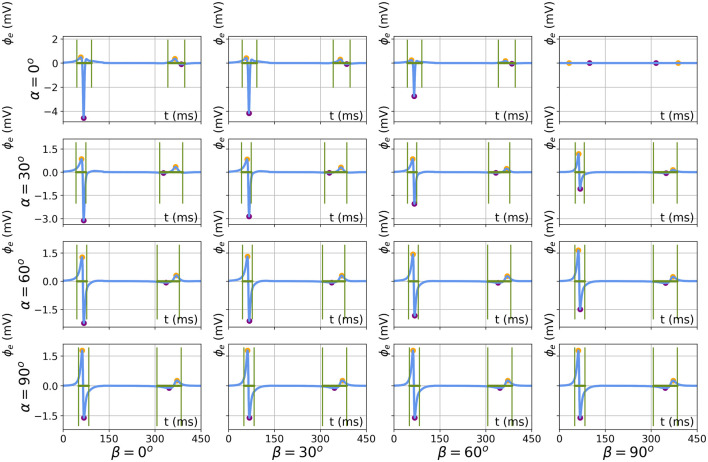
Results of feature extraction on numerically simulated electrograms for different catheter orientations (*α*, *β* ∈ [0°, 30°, 60°, 90°]). The results are shown for simulation parameters *L* = 5 mm, *d* = 2 mm, and *h* = 1 mm. Extracted features are the local maximum (orange), minimum (purple), and EGM width (green).

Figure 12 shows simulated bipolar signals for the different catheter orientations for a slab of thickness *L* = 5 mm. The minimum, maximum, peak-to-peak amplitude, and signal width were calculated as detailed in Section 2.4, in the same manner for simulated and analytical signals. In simulated signals, both the QRS complex and T-wave can be distinguished and the amplitude and width were also extracted for the T-wave.

#### 3.3.2 Effect of wall thickness

**FIGURE 13:**
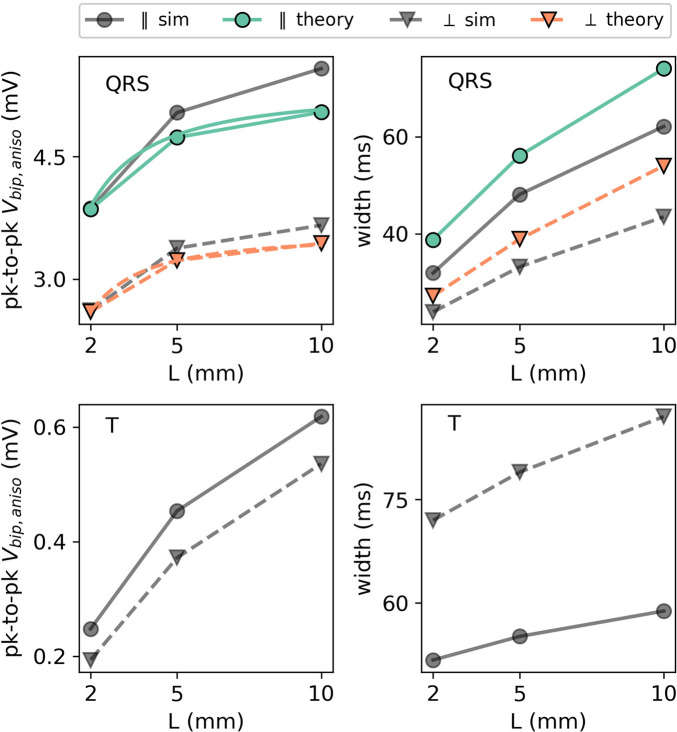
EGM properties as a function of wall thickness *L* for the extracellular potential in the two main directions: parallel (in the XY plane) and perpendicular (in the XZ plane) to the wave front. For the QRS part of the EGM signal, the theoretical prediction is plotted using colored lines. The different orientations are depicted with different markers. Every colored line (anisotropic theory) should be compared to the corresponding gray line (simulation) with the same marker and line style.

Electrogram amplitude and width are shown as a function of myocardial wall thickness in Figure 13. For the QRS complex, there is a good agreement between (full anisotropic) the theory and simulations.

We first observe that thicker walls yield larger iEGM amplitudes, both for the QRS complex and T-wave. This is in line with the solid angle theory and the exact solution in this paper; each unipolar signal arises as a difference between an endocardial and epicardial contribution. For thicker walls, the epicardial contribution decreases, such that the recorded amplitude will saturate at the amplitude of the unipolar signal originating from the endocardium.

Second, the width of the QRS complex also increases with the wall thickness. The reason is the same as for the amplitude: in the limit of *L* → *∞*, only endocardial contributions to (Eq. 36) matter, and this profile has the largest width.

#### 3.3.3 Effect of the myofiber orientation relative to the wave propagation direction

**FIGURE 14:**
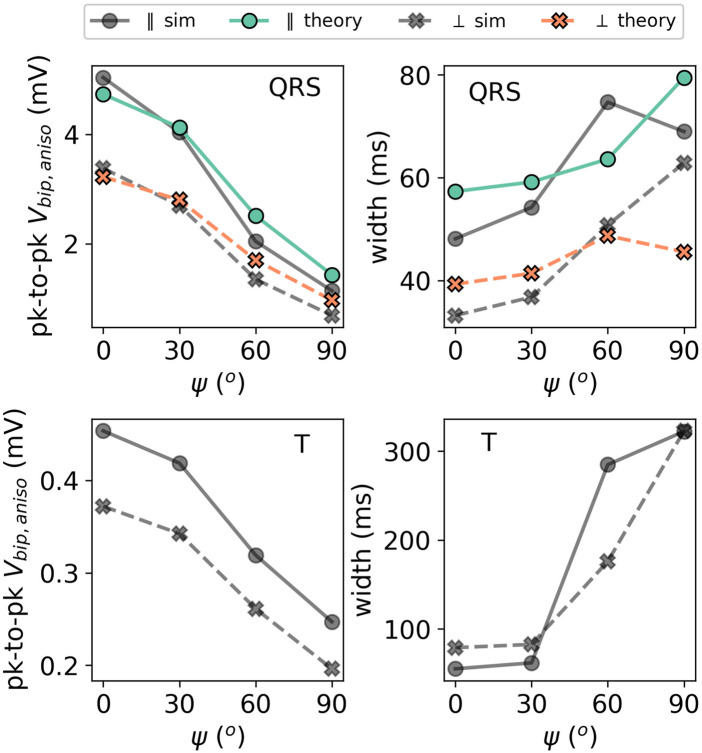
Illustration of the effect of the incidence angle *ψ* on the EGM properties for a slab with parallel fibers. The solid lines with circles as markers denote the direction parallel to the direction of wave propagation. The dashed lines with crosses as markers show the potential in the perpendicular direction. The colored lines (simulation output) should be compared to their corresponding gray lines (anisotropic theory) with the same marker and line style.

The effect of the myofiber direction on the amplitude and width of bipolar iEGMs is shown in Figure 14.

The amplitude is the highest for waves propagating along fibers and decreases for non-zero fiber angle *ψ*. This effect was already visible in Figure 9. From (Eq. 31), we can see that anisotropy affects the EGM amplitude in two different ways: via changing the effective tissue thickness and, hence, the solid angle and via the prefactor *A* of the leading order term (Eq. 32). By plotting hypothetical EGMs with one factor being left out, we found that the change in the amplitude is mostly caused by prefactor *A*(*ψ*) from Eq. 32 and only in a limited manner by re-scaled solid angles.

The myofiber orientation also affects the width of the measured EGMs. From (Eq. 32), we learn that two effects occur. First, increasing *ψ* decreases effective wall thickness *ℓ*, which leads to a less wide EGM, see paragraph Section 3.3.2. Second, the wave speed will decrease for larger *ψ*, as the wave does not propagate along the myofiber direction anymore. This effect will increase the EGM width. From Figure 14, we conclude that the reduction of the propagation velocity is the dominant effect, increasing the EGM width in case of a propagation transverse to the myofiber direction.

It can be further observed in Figure 9 that the theoretically predicted width of the QRS complex for *ψ* = 90° shows an outlier. The corresponding EGM was shown in Figure 9 (middle row, rightmost column). Due to the small signal amplitude, the width is determined by wide positive lobes, which makes the calculation of the width sensitive to small deviations.

## 4 Discussion

### 4.1 Relation to previous electrogram calculations

The morphology and properties of the electrograms depend on many confounding factors (De Bakker, 2019). Several studies have already investigated the impacts of various factors on EGMs or ECGs. For example, the influence of epicardial fat on uni- and bipolar electrogram amplitudes was studied in the following:van Huls van Taxis et al., 2013 and De Bakker, 2019. Wiley et al. (2005) and De Bakker (2019) investigated the influence of the electrode size on the EGM properties and found that the smaller the diameter, the steeper the EGM. De Bakker (2019) also highlights the importance of the catheter orientation and location for the correct interpretation of bipolar signals, while Ikeda et al. (2014) focused on the effect the contact force has on the morphology of the electrogram. Therefore, it is crucial to fundamentally understand the local electrogram in order to properly deduce the information from catheter mapping (Choudhuri and Akhtar, 2012).

Concerning the *ab initio* interpretation of electrograms, major progress was reported in the 1960s (Bayley and Berry, 1964; Gelernter and Swihart, 1964; Selvester et al., 1967), based on a solution of Poisson’s equation in a homogeneous medium. Under this assumption, the problem is equivalent to classical potential problems in electrostatics and gravity, enabling the expression of the measured potential as being proportional to the angle under which the electrode sees the electrical source (Plonsey, 1965; 1974; Holland and Arnsdorf, 1977; Macfarlane et al., 2010). This so-called solid angle theory was successful in explaining the overall signal shape, sharpness, and the influence of local infarcts, i.e., locally unexcitable tissue. Plonsey and Barr (1987) derived the potentials inside a half-space of the myocardium with parallel fibers using the method of mirrors, but provide no expression for the resulting electrograms measured in the blood pool. Van Oosterom (2002) specified that the electrogram equals the time course of the difference of two solid angles, which are maximally close to the surface with a maximum value of 2*π* or 360°. In addition, this concept allowed understanding that the interfaces in the medium, delineating regions with different conductivities, contribute to a large extent to the electrogram signal (Holland and Arnsdorf, 1977).

Regarding the ECG registered on the body surface (rather than the iEGM), it was argued that solid angle theory serves as a rational basis for understanding the ischemic TQ-ST deflection (Richeson et al., 1978). However, in order to explain the body-surface ECG, the inhomogeneous conductivity in the torso due to the bones, air, and the lungs and the finiteness of the tissue, needs to be taken into account, rendering the mathematical problem extremely complex, such that calculating accurate ECGs requires numerical methods (Pezzuto et al., 2017).

In recent years, the focus has shifted from studying electrograms analytically to doing numerical studies in order to understand and predict iEGMs. *In silico* studies were carried out by Blauer et al. (2014) and Gaeta et al. (2020, 2021) to figure out the effect of directionality and electrode spacing on bipolar amplitudes. Gaeta et al. derived a theoretical model based on local activation times (LATs) and validated it with clinical data. Jacquemet et al. (2003) used computer simulations to link the signal properties of unipolar EGMs to the underlying tissue during atrial fibrillation. Nonetheless, in this work, we argue that the theory for iEGMs can be extended by an analytical solution for the case of a slab with parallel myofibers and that these insights help understand the EGM amplitude and shapes.

In addition, there is a recurring question on whether given tissue parameters are better measured using a single electrode (unipolar signal), two nearby electrodes (bipolar signal), or a multi-electrode array (Leshem et al., 2017). Furthermore, electrodes of a normal size or micro-electrodes can be used (Berte et al., 2020; Glashan et al., 2021). Another question is whether it is better to use contact electrodes or a central basket within the blood pool (Narayan et al., 2012; Berte et al., 2020). Berte et al. (2020) outlines how physicians should be careful in interpreting voltages in electro-anatomical mapping procedures, since the electrode size and configuration has a significant impact on measured voltages and leads to inaccurate substrate detection and mapping.

The aforementioned questions have been partially addressed in theoretical studies, which generally did not include anisotropic wave propagation. Holland and Arnsdorf (1977) showed with the solid angle theory that the EGM properties change depending on the electrode location and the geometry of the infarcted tissue. Recent studies have argued that local potential differences, as observed by a bipolar electrode, or any pair in an array configuration, can be described as projections of an electrical field vector (Vermoortele et al., 2023). The effect of the catheter orientation (and, thus, the bipolar electrodes) on iEGMs has been addressed in the study by Gaeta et al. (2020), and the effects of anisotropy on the potential field were studied in Colli Franzone et al., 1982. Colli Franzone et al. (2000) further decomposed the dipole source to the EGM into a component along the myofiber and one along the normal wave front, but gave no explicit expression for the potentials to be measured.

### 4.2 Analytical solution for electrograms generated in a slab with parallel myofibers

#### 4.2.1 Agreement and differences with the solid angle theory

In this work, we derived the iEGM-shape from bidomain model equations, with increasing accuracy in the approximations: a homogeneous medium, a three-layered medium, and a three-layered medium with anisotropy. In earlier works, mirror sources have been applied in cylindrical or spherical heart and torso geometries (Bayley and Berry, 1964; Gelernter and Swihart, 1964; Selvester et al., 1967) to obtain insights on the body-surface ECG and on a two-layered myocardium with parallel fibers (Plonsey and Barr, 1987), to mimic recordings of electrodes placed within the tissue. Here, we focused on intracardiac EGMs measured in the blood pool as used in the clinic, requiring the inclusion of anisotropy. Figures 9, 14 show that the effect of anisotropy can indeed cause a 3.6-fold change in the amplitude of a bipolar signal.

The classical solid angle theory (Geselowitz, 1989; Van Oosterom, 2002) can be applied here for the case of equal conductivities (without myofibers), but fails for the anisotropic slab model. Figure 5 shows how the arctan function can be seen as the angle (in 2D and as a solid angle in 3D) from the electrode position toward the dipole sheet. In three-layered theories, reflections (mirror sources) also need to be taken into account.

#### 4.2.2 Nature of the singularity near electrical sources of the iEGM

Our analytical results also confirm that there are no points of infinite potential (singularities) when measuring close to a cardiac depolarization wave. This fact has also been recognized by others [e.g., Holland and Arnsdorf (1977)] using the solid angle theory, but we, nevertheless, prefer to repeat the arguments here.

A classical result in electromagnetism states that the potential near an electrical dipole scales as 1/*R* ^2^. This reasoning seems to suggest that there is a singularity (infinite value) of the potential on the myocardial surface (i.e., for *R* → 0). Such a result would clearly be non-physical. The flaw in the argumentation is that if the measurement electrode is put next to the endocardium (where *R* = 0), the dipole charge is distributed along the wave front; if the depolarization front has constant surface density charge *σ* = *p*/*A* (with *p* as the local dipole moment and *A* as the surface), then in a region of radius *R* around the electrode, a total dipole moment $P=\sigma \frac{\pi R^{2}}{2}$ is present, leading to a potential proportional to $P/R^{2}=\sigma \frac{\pi }{2}$ , which is finite. The absence of singularity in the potential is reflected in our analytical result, both in the homogenized case (Eq. 22) and the full anisotropic series solution (Eq. 31), see Figures 2, 4. The multi-valuedness of the arctan function accounts for both the discontinuity of Φ within the myocardial wall and the continuity of Φ outside it. The underlying mathematical object is a branch cut (Arfken and Weber, 1995), see also a recent application of this mathematical concept in cardiology as phase discontinuity and phase defects in the studies by Arno et al., 2021 and Tomii et al., 2021.

#### 4.2.3 Spatial decay rate

At large distances, we can find by which power law unipolar and bipolar voltages decay, using the Taylor series arctan(*ξ*) ≈ *ξ* − *ξ* ^3^/3 + ⋯ for small *ξ*. From the homogenized solution (Eq. 22), we have the following for an electrode at a large distance *h* = *R* from the endocardium:

$$\begin{array}{ll} {\tilde{\Phi }}_{\text{unipolar}}\left(t\right) & =\frac{\left(\phi_{max}-\phi_{rest}\right)g_{i,xx}}{2\pi \tilde{g}}\left(\frac{-vt}{R}-\frac{-vt}{R+L}\right)\\ & \;+O\left(R^{-3}\right)\propto \frac{L}{R^{2}}+O\left(R^{-3}\right). \end{array}$$ (40)

A similar argument is held for each term in the full anisotropic series solution. Thus, the unipolar signal decays as *R* ^−2^ with a distance from the myocardium *for large* *R*. Similarly, we find the following for parallel and perpendicular bipolar recordings:

$$V_{\text{bip},\|}\propto \frac{d}{R^{2}}+O\left(R^{-3}\right),\;V_{\text{bip},⊥}\propto \frac{d}{R^{3}}+O\left(R^{-4}\right).$$ (41)

The different decay law for both cases should be noted. This is rooted in the fact that holding the catheter perpendicular to the myocardial wall effectively takes the derivative with respect to the distance from myocardium (*R*), according to (Eq. 39). As the derivative of *R* ^−2^ is −2*R* ^−3^, this explains the cubic power decay of *V*_bip,⊥_. Both bipolar recordings are also proportional in the leading order to interelectrode distance *d*.

#### 4.2.4 Limitations of the analytical solution

The analytical solution presented here was designed for a planar wave front that remains perpendicular to endo- and endocardial boundaries. This is a simplification, as the correct boundary condition to *V*_*m*_, i.e., (Eq. 2j), will cause a V-shaped wave front due to bath loading (Roth, 1991). This effect is small in the studied regime, see Figure 2C. Future works should also address the bath-loading effect, which requires the inclusion of the wave front shape into the full PDE solution, which may be possible using our present results. Other extensions could, e.g., address rotational anisotropy and spatially varying wall parameters, such as anisotropy, conduction velocity, thickness, and repolarization.

### 4.3 Effects of myocardial and catheter properties on iEGM characteristics

#### 4.3.1 Influence of the wall thickness

A clinical study by Glashan et al. (2018) suggests that it is important to account for the wall thickness when analyzing electro-anatomical voltage mapping data. A linear dependence of bipolar and unipolar voltages on the wall thickness was proposed. Our results agree with the positive dependence of voltage on thickness; however, the exact dependence is more complicated than being linear, see (Eq. 35). In Figure 13, we see that the amplitude increases with increasing *L* both for the QRS complex and T-wave. For large *L*, the epicardium has a minimal influence and the difference becomes low; therefore, we see a saturation in *V*_bip_(*L*) curves, i.e., a horizontal asymptote. The bipolar electrograms observed at a position are in leading orders a difference of two contributions determined by the intersection of the propagation wave front with the boundaries of the medium. In summary, the addition of anisotropy and a three-layered medium does not qualitatively change the explanation given by the solid angle theory (Holland and Arnsdorf, 1977).

#### 4.3.2 Effect of the catheter orientation in bipolar signals

From the analytical solution for the unipolar voltage, a difference can be taken into account to obtain bipolar voltages. These signals vary in polarity between the parallel measurements to the wave front and are perpendicular to the wall. The bipolar signals measured at intermediate angles interpolate between these extremes and exhibit neither an even nor odd symmetry in time.

#### 4.3.3 Effect of the local myofiber orientation

Anisotropy of the cardiac wall acts in different manners on iEGMs: by changing the conduction velocity and by deflecting electrical field lines at the myocardial boundary, differently from the isotropic case. The latter effect causes a different apparent tissue thickness *ℓ* and also affects the prefactor in our formula (Eq. 31). In Figure 9, it is clear that *ψ* has a pronounced effect and the amplitude decreases approximately threefold if the wave is not propagating parallel but is rather perpendicular to the myofibers. We conclude that if clinicians want to use a threshold on the unipolar or bipolar amplitude to delineate viable tissue regions, not only the wall thickness, but also the local myofiber orientation (relative to the wave propagation) should be taken into account.

## 5 Conclusion

In this work, an analytical description of the intracardiac electrogram was presented for an anisotropic slab model of the myocardial wall, in which bath-loading effects are neglected. Including different conductivities in subdomains surpasses the classical solid angle theory as mirror images are present. However, for a recording electrode near the myocardium, the leading order term offers a reasonable qualitative description. In addition, the non-linear dependency of the EGM amplitude on the wall thickness is given. If the wave propagates perpendicular rather than parallel to the myofibers, the EGM amplitude is significantly reduced (due to conductivity effects) and its width increases (due to reduced propagation velocity). These results could prove useful when interpreting electrical voltage maps and selecting threshold values for tissue characterization.

## Data Availability

The raw data supporting the conclusion of this article will be made available by the authors, without undue reservation.

## Footnotes

1 Here, we avoided the use of the inverse cotangent function, since for some authors and software it is defined as $\frac{\pi }{2}-\arctan\left(x\right)$ , while for others, it is arctan(1/*x*), and both definitions differ for *x* < 0.
