# Supplementary material for: Impact of electrode orientation, myocardial wall thickness, and myofiber direction on intracardiac electrograms: numerical modeling and analytical solutions
Source: Front Physiol. 2023 Jul 10;14:1213218. doi: 10.3389/fphys.2023.1213218 (PMC10364610; doi:10.3389/fphys.2023.1213218)
Supplement: Supplementary file 1 [file DataSheet1.pdf]

Supplementary Material:Impact of Electrode Orientation, Myocardial Wall Thickness and Myofiber Direction on Intracardiac Electrograms: Numerical Modeling and Analytical Solutions

A APPENDIX: THEORETICAL FRAMEWORK INCLUDING ANISOTROPY

A.1 EGM generated in an isotropic 3-layered medium

We considered a three-layered geometry consisting of torso, myocardium and blood pool. This medium has a piece-wise constant conductivity and a piece-wise defined potential:

$$g = \begin{cases} g_{\mathrm{T}} & (z < -L) \\ g_{\mathrm{M}} & (-L < z < 0) \\ g_{\mathrm{B}} & (0 < z) \end{cases}, \qquad \Phi = \begin{cases} \Phi_{\mathrm{T}} & (z < -L) \\ \Phi_{\mathrm{M}} & (-L < z < 0) \\ \Phi_{\mathrm{B}} & (0 < z) \end{cases}. \tag{S1}$$

As a function of time, the whole solution translates at constant speed v in the X-direction, such that we could work in a co-moving coordinate frame. The equations to solve became:

$$g_{\mathrm{T}}\partial_x^2\Phi_{\mathrm{T}} + g_{\mathrm{T}}\partial_z^2\Phi_{\mathrm{T}} = 0, \qquad (z < -L) \tag{S2a}$$

$$g_{\mathrm{M}}\partial_x^2\Phi_{\mathrm{M}} + g_{\mathrm{M}}\partial_z^2\Phi_{\mathrm{M}} = g_{\mathrm{i},xx}(\phi_{\mathrm{max}} - \phi_{\mathrm{rest}})\delta'(x)H(z+L)H(-z), \qquad (-L < z < 0) \tag{S2b}$$

$$g_{\mathrm{B}}\partial_x^2\Phi_{\mathrm{B}} + g_{\mathrm{B}}\partial_z^2\Phi_{\mathrm{B}} = 0, \qquad (0 < z), \tag{S2c}$$

$$\Phi_{\mathrm{T}} = \Phi_{\mathrm{M}}, \qquad (z = -L) \tag{S2d}$$

$$g_{\mathrm{T}}\partial_z\Phi_{\mathrm{T}} = g_{\mathrm{M}}\partial_z\Phi_{\mathrm{M}}, \qquad (z = -L) \tag{S2e}$$

$$\Phi_{\mathrm{B}} = \Phi_{\mathrm{M}}, \qquad (z = 0) \tag{S2f}$$

$$g_{\mathrm{B}}\partial_z\Phi_{\mathrm{B}} = g_{\mathrm{M}}\partial_z\Phi_{\mathrm{M}}. \qquad (z = 0) \tag{S2g}$$

As in the homogenized case, we took one step back and replaced the source by a monopole line charge at $x = 0$, $z = -R$. This line charge is located within the myocardium, thus $R \in (0, L)$. We thus temporarily replaced (S2b) by

$$g_{\mathrm{M}}\partial_x^2\phi_{\mathrm{M}} + g_{\mathrm{M}}\partial_z^2\phi_{\mathrm{M}} = -b_0\delta(x)\delta(z+R). \qquad (-L < z < 0) \tag{S3}$$

In the method of mirror images, the field in each subdomain is written as a sum of elementary charges in the other domains, which are located at the same distance from the interface as the original charge, see Fig. S1. We denoted the point charge potential (Green's function) in a 2D medium and its derivative as

$$F(x, z; z_0) = -\frac{1}{4\pi} \ln[x^2 + (z - z_0)^2], \tag{S4}$$

$$\partial_z F(x, z; z_0) = -\frac{1}{2\pi} \frac{z - z_0}{x^2 + (z - z_0)^2}. \tag{S5}$$

If the original source is not lying at the middle of the slab, the source point is reflected around each of the bounding planes, generating multiple images, see Fig. S1. If z_0 is the location of the true source, then the mirror sources are found at:

$$z_j = \begin{cases} j\ell - R & (j \text{ even}) \\ (j-1)\ell + R & (j \text{ odd}) \end{cases}. \tag{S6}$$

Following (Jackson, 1975; Escribano et al., 2017), we constructed solutions of the form:

$$\begin{aligned} \Phi_{\mathrm{T}}(x, z) &= \sum_{j=0}^{\infty} \frac{a_j}{g_{\mathrm{T}}} F(x, z, z_j), \\ \Phi_{\mathrm{M}}(x, z) &= \sum_{j=-\infty}^{\infty} \frac{b_j}{g_{\mathrm{M}}} F(x, z, z_j), \\ \Phi_{\mathrm{B}}(x, z) &= \sum_{j=-\infty}^{0} \frac{c_j}{g_{\mathrm{B}}} F(x, z, z_j), \end{aligned} \tag{S7}$$

with unknown coefficients a_j, b_j, c_j (and $b_0 = g_{\mathrm{i},xx}(\phi_{\max} - \phi_{\mathrm{rest}})$ known). Note that the image sources can only appear outside the subdomain where the expansion is used. Therefore, all a_j for negative j and all c_j for positive j have been set equal to 0. By construction, this solution already obeys Eqs. (S2a),(S2c) and (S3) in the bulk. Next, we imposed the joint conditions (S2f), (S2g) at the myocardium-blood interface ($z = 0$):

$$\sum_{j=-\infty}^{\infty} \frac{b_j}{g_{\mathrm{M}}} F(x, 0, z_j) = \sum_{j=-\infty}^{0} \frac{c_j}{g_{\mathrm{B}}} F(x, 0, z_j), \tag{S8}$$

$$\sum_{j=-\infty}^{\infty} b_j \partial_z F(x, 0, z_j) = \sum_{j=-\infty}^{0} c_j \partial_z F(x, 0, z_j). \tag{S9}$$

Both sides of these equations depend on the continuous variable x. Therefore, to satisfy them, terms with the same relative distance to the interface on both sides should cancel out. E.g. the mirror sources at a distance R from $Z = 0$ should on their own fulfill the equality, such that, for all x:

$$\frac{b_0}{g_{\mathrm{M}}} F(x, 0, -R) + \frac{b_1}{g_{\mathrm{M}}} F(x, 0, R) = \frac{c_0}{g_{\mathrm{B}}} F(x, 0, -R), \tag{S10}$$

$$b_0 \partial_z F(x, 0, -R) + b_1 \partial_z F(x, 0, R) = c_0 \partial_z F(x, 0, -R). \tag{S11}$$

Now, since F is even in $z - z_0$ and $\partial_z F$ is odd in $z - z_0$, this results in

$$\frac{b_0 + b_1}{g_\mathrm{M}} = \frac{c_0}{g_\mathrm{B}}, \qquad b_0 - b_1 = c_0, \tag{S12}$$

which can be readily solved to

$$b_1 = \frac{g_\mathrm{M} - g_\mathrm{B}}{g_\mathrm{M} + \eta g_\mathrm{B}} b_0 := h_\mathrm{B} b_0, \qquad c_0 = \frac{2g_\mathrm{B}}{g_\mathrm{M} + g_\mathrm{B}} b_0 := v_\mathrm{B} b_0. \tag{S13}$$

Here, we have defined the 'horizontal' and 'vertical' transfer operators h_B and v_B, referring to the directions as in Fig. S1: 'horizontal' means finding a coefficient in another layer (towards B or towards T), while 'vertical' means finding a blood or torso coefficient from the same coefficient in the myocardium domain.

We repeated this calculation at the torso-myocardium interface (at $z = -L$):

$$b_{-1} = \frac{g_\mathrm{M} - g_\mathrm{T}}{g_\mathrm{M} + g_\mathrm{T}} b_0 := h_\mathrm{T} b_0, \qquad a_0 = \frac{2g_\mathrm{T}}{g_\mathrm{M} + g_\mathrm{T}} b_0 = v_\mathrm{T} b_0. \tag{S14}$$

In both cases, the proportionality constants representing a horizontal (h) or vertical (v) jump in Fig. S1 are

$$h_* = \frac{g_\mathrm{M} - g_*}{g_\mathrm{M} + \eta g_*}, \qquad v_* = \frac{2g_*}{g_\mathrm{M} + g_*}, \tag{S15}$$

extending the results in (Jackson, 1975; Escribano et al., 2017) to a 3-layer set-up with unequal conductivities in the outer layers. Next, we took the (mirrored) sources at a distance $L + R$ from the interface $z = 0$:

$$b_{-1} + b_2 = c_{-1}, \tag{S16}$$

$$g_\mathrm{M}[b_{-1} - b_2] = g_\mathrm{B} c_{-1}. \tag{S17}$$

This system is identical to (S12), whence

$$b_2 = h_\mathrm{B} b_{-1} = h_\mathrm{B} h_\mathrm{T} b_0, \qquad c_{-1} = v_\mathrm{B} b_{-1} = v_\mathrm{B} h_\mathrm{T} b_0. \tag{S18}$$

From the sources at a distance $2L - R$ from $z = -L$, we found

$$b_{-2} = h_\mathrm{T} b_1 = h_\mathrm{T} h_\mathrm{B} b_0, \qquad a_1 = v_\mathrm{T} b_1. \tag{S19}$$

These steps lead to the following recursive scheme:

$$\begin{aligned}
b_j &= h_\mathrm{B} b_{-(j-1)}, && \text{(step to the right)}\\
b_{-j} &= h_\mathrm{T} b_{j-1}, && \text{(step to the left)}\\
a_j &= v_\mathrm{T} b_j, && \text{(step up)}\\
c_j &= v_\mathrm{B} b_j. && \text{(step down)}
\end{aligned} \tag{S20}$$

Solving this system leads for $n \in \mathbb{N}$ to $b_{\pm 2n} = h_\mathrm{B}^n h_\mathrm{T}^n b_0$, $b_{2n+1} = h_\mathrm{B}^{n+1} h_\mathrm{T}^n b_0$ and $b_{-2n-1} = h_\mathrm{T}^{n+1} h_\mathrm{B}^n b_0$, which can be summarized as

$$b_j = \begin{cases} h_\mathrm{T}^{\lfloor \frac{j}{2} \rfloor} h_\mathrm{B}^{\lceil \frac{j}{2} \rceil} g_{\mathrm{i},xx}(\phi_\mathrm{max} - \phi_\mathrm{rest}), & (j > 0) \\ h_\mathrm{B}^{\lfloor -\frac{j}{2} \rfloor} h_\mathrm{T}^{\lceil -\frac{j}{2} \rceil} g_{\mathrm{i},xx}(\phi_\mathrm{max} - \phi_\mathrm{rest}). & (j < 0) \end{cases} \tag{S21}$$

Here, we used the ceiling and floor operators for a compact notation.

Hence, the electrical potential originating from a line charge placed at $z = -R$, $x = 0$ within isotropic myocardium and measured in the blood pool equals

$$\Phi_\mathrm{B}^\mathrm{line}(x,z) = \frac{v_\mathrm{B}}{g_\mathrm{B}} \sum_{j=-\infty}^{0} b_j F(x,z,z_j) = \frac{v_\mathrm{B} g_{\mathrm{i},xx}(\phi_\mathrm{max} - \phi_\mathrm{rest})}{g_\mathrm{B}} \sum_{j=0}^{\infty} h_\mathrm{B}^{\lfloor \frac{j}{2} \rfloor} h_\mathrm{T}^{\lceil \frac{j}{2} \rceil} F(x,z,z_{-j}). \tag{S22}$$

To obtain the potential of a dipole layer extending over $[-L, 0]$, we differentiated with respect to x, added a minus sign and integrated over z_0. Recalling from the homogenized case that

$$-\int \frac{\partial}{\partial x} F(x,z;z_i) \mathrm{d}z_i = \frac{1}{2\pi} \arctan\left(\frac{z - z_i}{x}\right) + C, \tag{S23}$$

we applied these actions to each term in the series (S22) to find:

$$\Phi_\mathrm{B}(x,z) = \frac{v_\mathrm{B} g_{\mathrm{i},xx}(\phi_\mathrm{max} - \phi_\mathrm{rest})}{2\pi g_\mathrm{B}} \sum_{j=0}^{\infty} h_\mathrm{B}^{\lfloor \frac{j}{2} \rfloor} h_\mathrm{T}^{\lceil \frac{j}{2} \rceil} \left[\arctan\left(\frac{x}{Z + jL}\right) - \arctan\left(\frac{x}{Z + (j+1)L}\right)\right]. \tag{S24}$$

In the blood domain where the recording is taken, $Z = z$. Going back to the stationary frame of reference by replacing x with $x - vt$ yields (31) in the main text.

A.2 EGM generated in a 3-layered medium with constant myofiber direction

In the middle layer representing the myocardium, we had for the components of $\mathbf{G} = \mathbf{G}_\mathrm{i} + \mathbf{G}_\mathrm{e}$:

$$g_{xx} = g_{\mathrm{i},xx} + g_{\mathrm{e},xx} = (g_{\mathrm{i,l}} + g_{\mathrm{e,l}}) \cos^2 \psi + (g_{\mathrm{i,t}} + g_{\mathrm{e,t}}) \sin^2 \psi \tag{S25}$$

$$g_{zz} = g_{\mathrm{i,t}} + g_{\mathrm{e,t}}, \qquad g_{xz} = 0. \tag{S26}$$

Then, the bidomain equations became, in the co-moving frame:

$$
\begin{aligned}
g_\mathrm{T}\partial_x^2\Phi_\mathrm{T} + g_\mathrm{T}\partial_z^2\Phi_\mathrm{T} &= 0, && (z < -L) && \text{(S27a)}\\
g_{xx}\partial_x^2\Phi_\mathrm{M} + g_{zz}\partial_z^2\Phi_\mathrm{M} &= g_{\mathrm{i},xx}(\phi_\mathrm{max} - \phi_\mathrm{rest})\delta'(x)H(z+L)H(-z), && (-L < z < 0) && \text{(S27b)}\\
g_\mathrm{B}\partial_x^2\Phi_\mathrm{B} + g_\mathrm{B}\partial_z^2\Phi_\mathrm{B} &= 0, && (0 < z) && \text{(S27c)}\\
\Phi_\mathrm{T} &= \Phi_\mathrm{M}, && (z = -L) && \text{(S27d)}\\
g_\mathrm{T}\partial_z\Phi_\mathrm{T} &= g_{zz}\partial_z\Phi_\mathrm{M}, && (z = -L) && \text{(S27e)}\\
\Phi_\mathrm{B} &= \Phi_\mathrm{M}, && (z = 0) && \text{(S27f)}\\
g_\mathrm{B}\partial_z\Phi_\mathrm{B} &= g_{zz}\partial_z\Phi_\mathrm{M}. && (z = 0) && \text{(S27g)}
\end{aligned}
$$

Next, we performed a change of coordinates $Z(z)$ according to (29) and defined $\Phi(x, z) = \phi(x, Z)$. With the anisotropy factor $\eta = \sqrt{g_{xx} + g_{zz}}$, the re-scaled tissue thickness $\ell = \eta L$ and $g_{xx} = g_\mathrm{M}$, the PDE system became

$$
\begin{aligned}
g_\mathrm{T}\partial_x^2\phi_\mathrm{T} + g_\mathrm{T}\partial_Z^2\phi_\mathrm{T} &= 0, && (Z < -\ell) && \text{(S28a)}\\
g_\mathrm{M}\partial_x^2\phi_\mathrm{M} + g_\mathrm{M}\partial_Z^2\phi_\mathrm{M} &= g_{\mathrm{i},xx}(\phi_\mathrm{max} - \phi_\mathrm{rest})\delta'(x)H(Z+\ell)H(-Z), && (-\ell < Z < 0) && \text{(S28b)}\\
g_\mathrm{B}\partial_x^2\phi_\mathrm{B} + g_\mathrm{B}\partial_Z^2\phi_\mathrm{B} &= 0, && (0 < Z), && \text{(S28c)}\\
\phi_\mathrm{T} &= \phi_\mathrm{M}, && (Z = -\ell) && \text{(S28d)}\\
g_\mathrm{T}\partial_Z\phi_\mathrm{T} &= \frac{g_\mathrm{M}}{\eta}\partial_Z\phi_\mathrm{M}, && (Z = -\ell) && \text{(S28e)}\\
\phi_\mathrm{B} &= \phi_\mathrm{M}, && (Z = 0) && \text{(S28f)}\\
g_\mathrm{B}\partial_Z\phi_\mathrm{B} &= \frac{g_\mathrm{M}}{\eta}\partial_Z\phi_\mathrm{M}. && (Z = 0) && \text{(S28g)}
\end{aligned}
$$

This system differed only from the isotropic case in the junction conditions (S28e), (S28g), where g_M needed to be replaced by $\frac{g_\mathrm{M}}{\eta}$. This lead to modifications in the transfer coefficients ($h_* \to H_*$, $v_* \to V_*$), see (30) and an effective myocardial thickness in the resulting EGM expression (31).

A.3 Series solution for the bipolar iEGMs

By taking the difference between two unipolar electrodes, we found the following result for the bipolar voltage for arbitrary orientation, valid for $z > 0$:

$$
\begin{aligned}
V_\mathrm{bip}(\alpha, \beta, h, d, t) = -\frac{V_\mathrm{B} g_{\mathrm{i},xx}(\phi_\mathrm{max} - \phi_\mathrm{rest})}{2\pi g_\mathrm{B}} \sum_{j=0}^{\infty} H_\mathrm{B}^{\lfloor \frac{j}{2} \rfloor} H_\mathrm{T}^{\lceil \frac{j}{2} \rceil} \Bigg[\arctan\left(\frac{x_0 - vt}{h + (j+1)\eta L}\right) \\
- \arctan\left(\frac{x_0 - vt}{h + j\eta L}\right) + \arctan\left(\frac{x_0 + d\cos\beta\cos\alpha - vt}{h + d\sin\alpha + (j+1)\eta L}\right) - \arctan\left(\frac{x_0 + d\cos\beta\cos\alpha - vt}{h + d\sin\alpha + j\eta L}\right)\Bigg].
\end{aligned}
\tag{S29}
$$

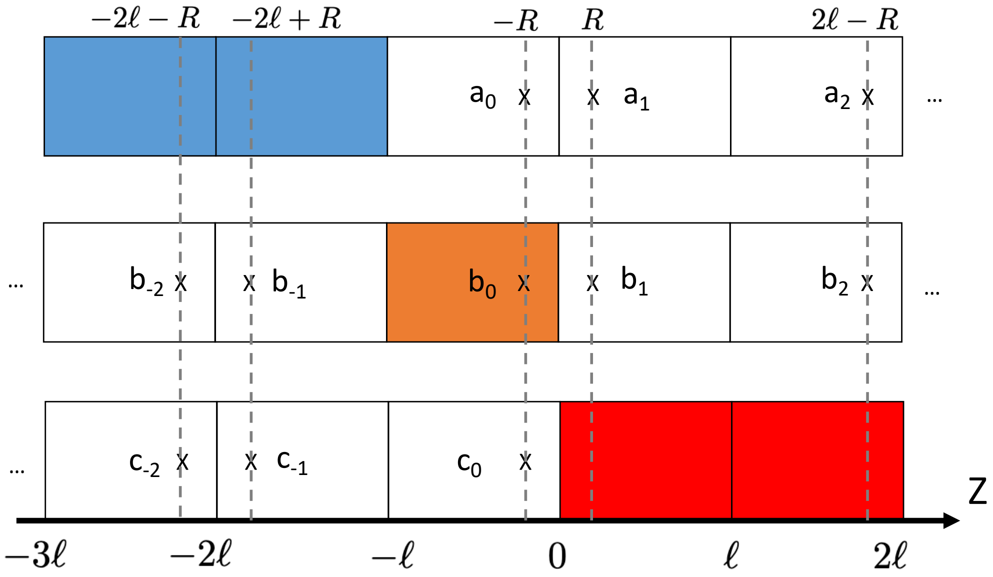

Figure S1: Overview of mirror sources, denoted by a cross, in the XZ-plane introduced to find an analytical EGM in a three-layered isotropic medium. The mirror planes are $Z = j\ell$, and the mirror sources are located at $Z_j, j \in \mathbb{Z}$ given by (S6). Top row: the torso subdomain (blue) requires mirror sources a_j for positive integer j. Middle row: the myocardial subdomain (orange) requires mirror sources b_j for integer $j \neq 0$, where b_0 is the original source within the depolarization wave. Bottom row: the blood pool subdomain (red) requires mirror sources c_j, for negative integer j.

REFERENCES

Escribano, S., Yeyati, A., and Prada, E. (2017). Interaction-induced zero-energy pinning and quantum dot formation in Majorana nanowires. *Beilstein Journal of Nanotechnology* 9. doi:10.3762/bjnano.9.203

Jackson, J. (1975). *Classical Electrodynamics* (New York: John Wiley & Sons), 2nd edn.
